# Differential Expression of HERV-K (HML-2) Proviruses in Cells and Virions of the Teratocarcinoma Cell Line Tera-1

**DOI:** 10.3390/v7030939

**Published:** 2015-03-04

**Authors:** Neeru Bhardwaj, Meagan Montesion, Farrah Roy, John M. Coffin

**Affiliations:** 1 Graduate Program in Molecular Biology and Microbiology, Sackler School of Graduate Biomedical Sciences, Tufts University, 136 Harrison Avenue, Boston, MA 02111, USA; E-Mail: neeru.bhardwaj@tufts.edu; 2 Graduate Program in Genetics, Sackler School of Graduate Biomedical Sciences, Tufts University, 136 Harrison Avenue, Boston, MA 02111, USA; E-Mails: meagan.montesion@tufts.edu (M.M.); farrah.roy@tufts.edu (F.R.); 3 Department of Molecular Biology and Microbiology, Tufts University, 136 Harrison Avenue, Boston, MA 02111, USA

**Keywords:** HERV-K (HML-2), endogenous retrovirus, gene expression, next-generation sequencing

## Abstract

Human endogenous retrovirus (HERV-K (HML-2)) proviruses are among the few endogenous retroviral elements in the human genome that retain coding sequence. HML-2 expression has been widely associated with human disease states, including different types of cancers as well as with HIV-1 infection. Understanding of the potential impact of this expression requires that it be annotated at the proviral level. Here, we utilized the high throughput capabilities of next-generation sequencing to profile HML-2 expression at the level of individual proviruses and secreted virions in the teratocarcinoma cell line Tera-1. We identified well-defined expression patterns, with transcripts emanating primarily from two proviruses located on chromosome 22, only one of which was efficiently packaged. Interestingly, there was a preference for transcripts of recently integrated proviruses, over those from other highly expressed but older elements, to be packaged into virions. We also assessed the promoter competence of the 5’ long terminal repeats (LTRs) of expressed proviruses via a luciferase assay following transfection of Tera-1 cells. Consistent with the RNASeq results, we found that the activity of most LTRs corresponded to their transcript levels.

## 1. Introduction

The genomes of all mammals, and indeed of most or all vertebrates, contain sequences of retroviral origin. Retroviruses have the unique ability to convert their ssRNA genome into dsDNA, which is then irreversibly integrated, as a provirus, into the host genome as part of the replication cycle. Though infection generally occurs through horizontal transfer, where a retrovirus infects a somatic cell, replicates and is passed from cell to cell and from one individual to another, proviruses resulting from infection of germline cells can also be inherited and transferred vertically, from parent to offspring [1]. Human endogenous retroviruses (HERVs) are the vestiges of infection of the germline cells of our ancestors and comprise ~8% of the genome [2]. Once integrated into the genome, HERVs are inherited in a Mendelian fashion, akin to genes, and subject to similar selection pressures, as insertions can have beneficial, detrimental or neutral effects on a host [3].

One group of HERVs, called HERV-K (HML-2), includes >90 proviruses and ~950 solo long terminal repeats (LTRs), produced from recombination between the 5’ and 3’ LTRs of a provirus [4]. Some HML-2 insertions integrated into the genome after the human-chimpanzee split and are the only HERVs specific to the human lineage [5]. At least 11 of these proviruses are still insertionally polymorphic within the human population and the question of their continued integration into the human germline remains open [5,6,7]. Interestingly, many proviruses in the HML-2 group retain close to full-length genomic sequence and some have maintained open reading frames for the retroviral genes *gag*, *pro*, *pol* and *env* [5].

HML-2 (Human MMTV-like, group 2) proviruses were named for the similarity of their *pol* sequence to mouse mammary tumor virus (MMTV), the viral cause of mammary carcinoma in mice [8,9,10]. Correspondingly, HML-2 expression has been linked to numerous disease states in humans. HML-2 expression in humans was first clearly linked to teratocarcinoma, where HML-2 RNA, protein and non-infectious virions are produced from diseased cells [11,12,13,14] and patients exhibit immune responses against expressed HML-2 antigens [15,16,17]. Remarkably, new types of spliced transcripts encoded by HML-2 were discovered in teratocarcinoma cells, later named *rec* and *np9* [18]. Rec is functionally analogous to HIV-1 Rev in shuttling unspliced or partially spliced mRNA out of the nucleus into the cytoplasm and is encoded by proviruses that were integrated with full-length sequence, called type 2 HML-2 proviruses [19]. Conversely, Np9 has no known function in the HML-2 replication cycle. In fact, *np9* is the result of a 292-bp deletion at the *pol*-*env* boundary in a contingent of defective proviruses, referred to as type 1 HML-2 proviruses, where the deletion creates a new splice donor site [20]. In addition to teratocarcinoma, HML-2 expression is often observed in other cancers, including breast cancer [21,22,23,24,25,26] and melanoma [27,28], and during HIV-1 infection [29,30,31,32,33,34]. However, a causal role for HML-2 proviral expression in human disease has not yet been identified.

A potential hurdle to examining the effect of HML-2 expression on the human host is determining which of the multiple HML-2 proviruses are active in different disease states. PCR approaches can reliably detect HML-2 RNA transcripts, however may not be able to discriminate among all the individually expressed HML-2 proviruses. In terms of pathogenic potential and association with disease, the proviral source of HML-2 expression is likely important because of their varying sequence preservation and coding potential [5]. In addition, due to their recent integration, accurate detection of many of the evolutionarily young HML-2 integrations is challenging as they are remarkably similar in sequence and finding unique regions to amplify may not be straightforward for each provirus. Due to sequence similarity, PCR recombination may pose a threat to accurate detection of individual proviruses if more than one is expressed at a time. Gold standard PCR methods like single genome sequencing [35] can effectively circumvent most issues, however amplified targets will be limited by the primer design of the assay and the throughput of the method.

High throughput next-generation sequencing approaches like RNASeq have been used to quantify expression of specific proviruses belonging to older groups of HERVs, including HERV-H [36] and HERV-W [37], and more recently have been applied to the HML-2 group [38,39]. Because of their more recent integration into the human genome, assignment of sequence reads to specific HML-2 proviruses is more difficult. Here, we use RNASeq to quantify expression of the more recently integrated HML-2 proviruses in the human teratocarcinoma cell line Tera-1 and in the virions it produces. As mentioned previously, teratocarcinoma cells are unusual in that they express HML-2 RNA and protein and also produce virions, a phenomenon that has only been reliably identified in a few other cell types [28,40], and the mechanism by which they do so has been largely unexplored [13,41]. By using a bioinformatic approach that calculates expression levels based solely on unique alignments, similar to a previous approach [39], we identified a number of distinct HML-2 proviral transcripts expressed in Tera-1 cells, including both evolutionarily older and younger elements. Two of the most highly expressed proviruses are present on chromosome 22, and closer analysis reveals distinct mechanisms of transcription for each provirus. In addition, promoter activity assays performed using the 5’ LTRs of expressed proviruses corroborate RNASeq results, demonstrating LTR function. Interestingly, only transcripts of the younger HML-2 elements appear to be packaged in Tera-1 virions, even though both old and young proviruses are expressed in the cell. This result implies that a selective process, potentially reliant on a packaging signal, is occurring for some of the more preserved HML-2 proviruses, supporting an observation made previously [13]. Thus, RNASeq analysis can effectively discriminate HML-2 provirus expression profiles and can be used to uncover basic features of HML-2 biology.

## 2. Materials and Methods

### 2.1. Cell Culture

The human teratocarcinoma cell line Tera-1 (ATCC, Manassas, VA, USA, Cat# HTB-105) was grown in McCoy’s 5A media (Life Technologies, Carlsbad, CA, USA, Cat# 16600-082), supplemented with 15% FBS (Atlanta Biologicals, Norcross, GA, USA, Cat# S11195) and 1% Pen-Strep (Life Technologies Cat# 15140-122). Feline astrocyte G355.5 cells (ATCC Cat# CRL-2033) were grown in McCoy’s 5A media supplemented with 10% FBS and 1% Pen-Strep. 293T cells (ATCC Cat# CRL-2316) were grown in DMEM supplemented with 10% FBS and 1% Pen-Strep. All cell lines were grown at 37 °C with 5% CO_2_.

### 2.2. RNA Extraction

Passage-matched Tera-1 cells and Tera-1 supernatant were collected from 100 mm cell culture plates. Culture supernatant was spun down for 5 min at 1200× *g* and 0.22 μm filtered to remove cellular debris. 1 mL of 0.25% Trypsin-EDTA (Gibco, Carlsbad, CA, USA, Cat# 25200-056) was added to the cell culture plate in order to remove cells and incubated at 37 °C until detached. Cells were removed from the plate, washed once with 5 mL of 1× Phosphate-buffered saline (PBS; Gibco Cat# 14190), and pelleted for 5 min at 1200× *g*. Dry pellets of Tera-1 cells and filtered supernatant samples were frozen at −80 °C until the extraction procedures were performed. For RNA extraction, 1–2 million Tera-1 cells were used as input for the TRIzol-PureLink RNA system (Ambion, Carlsbad, CA, USA, Cat# 15596-026 and Cat# 1218301A). Virions were pelleted at 21,000× *g* from 3 mL of cell supernatant and RNA was extracted using guanidinium isothiocyanate (Sigma, St. Louis, MO, USA, Cat# 50983), as described previously [34]. Tera-1 cellular RNA was treated with 2U DNase (Ambion, Turbo DNA-free kit, Cat# AM1907) for 1 h at 37 °C and virion RNA was treated with 1.5U of DNase for 45 min at 37 °C. RNA was confirmed to lack detectable DNA by performing a quantitative PCR for detection of HML-2 template, where DNA contamination is evident via amplification in wells lacking reverse transcriptase. This qPCR has been described previously [34].

### 2.3. RNASeq Library Preparation

An Illumina RNASeq library was prepared from the Tera-1 cell RNA using the TruSeq Stranded Total RNA kit with Ribo-Zero Gold (Illumina, San Diego, CA, USA, Cat# RS-122-2301), which removes ribosomal RNA from the test sample. ~1 ug of Tera-1 RNA was depleted of rRNA and resulting RNA was incubated at 65 °C for 5 min to avoid shearing (as recommended in alternate protocol), which should produce cDNA fragments ranging from 130–350 bases in length due to random priming. Our RNA fragments showed an average length of 190 bases using BioAnalyzer (Agilent Technologies, Santa Clara, CA, USA) peak analysis. This step was followed by reverse transcription, end repair, an A-tailing reaction to add a single 3’ A-overhang to the fragments and then ligation of barcoded sequencing adaptors with a T-overhang to bind these fragments. The library was amplified using adaptor-specific primers for 10–15 cycles of PCR. An Illumina RNASeq library was prepared from the Tera-1 virion RNA using the NuGen Ovation v2 kit (NuGen, San Carlos, CA, USA, Part# 7102). This kit does not allow for strand-marking (dUTP incorporation) during cDNA synthesis. It takes low input samples like virion RNA and amplifies RNA using a proprietary process. Amplified RNA is converted to cDNA and the cDNA is sheared using a targeted sonicator (Covaris, Woburn, MA, USA, M-Series, M220). Virion cDNA was sheared to 200–600 bp, as determined using a BioAnalyzer. The Tera-1 virion library was prepared from the cDNA using end repair, A-tailing, barcoded adaptor ligation and library amplification as described above. Both libraries were run together on the MiSeq benchtop sequencer (Tera-1 cell library = 95% input; Tera-1 virion library = 5% input) using the v3 kit that allows for paired-end (PE) reads up to 301 bases in length (Illumina, Cat# MS-102-3001). 26 million PE reads were generated for the Tera-1 cell library and 1.2 million PE reads for the Tera-1 virion library.

### 2.4. RNASeq Analysis

MiSeq reads from the Tera-1 cell and virion libraries were trimmed to remove Illumina adaptor sequences, low quality reads (Q < 25) and reads shorter than 100 bases using the program Trimmomatic [42]. Trimmed paired-end reads from the Tera-1 cell and virion libraries were aligned to the hg19 build of the human genome or to a faux “HML-2 genome” which is a FASTA file containing the sequences of 93 proviruses (4 are present only as solo LTRs in hg19, and 2 are present as pre-integration sites in hg19) and 943 solo LTRs. Both alignments were performed using TopHat v2.0.10, which used Bowtie v2.1.0 as the underlying aligner [43,44] and allowed for up to 2 mismatches to a mapping location for unique or multi-mapped reads. Hg19 alignments for the Tera-1 cell reads were performed using the –fr-firststrand option which allows for the strandedness of the read to be incorporated into the alignment data (“Plus stranded”) or without with this option (“Unstranded”). Output .bam files from the alignment were either (1) sorted and kept unfiltered (“Unfiltered”) which retains reads that align to multiple targets as well as those that uniquely align to a single provirus or (2) sorted and filtered for uniquely aligned reads (“Unique Only”) using SAMtools [45]. TopHat2 assigns uniquely aligned reads a mapping quality (MAPQ) score of 50 and these reads can be selected for from the total alignment using the SAMtools view –q 50 command. Aligned reads from the cell library had an average insert size of 180 bases (range: 98–522) as compared to 200 bases (range: 100–568) for the virion library, which was determined using Picard Tools (Broad Institute) for QC analysis of the .bam files.

Cufflinks v2.2.1 [46] was used to generate estimates of transcript abundance normalized to the length of the expressed gene, outputted as fragments per kilobase per million mapped reads (FPKM). Hg19 transcript annotation files (GTF format) contained annotations for 87 HML-2 full proviruses, 4 proviruses present only as solo LTRs in hg19 and 947 solo LTRs in addition to cellular transcripts. HML-2 genome GTF files contained annotations for all 93 included proviruses and 943 solo LTRs. Cufflinks was run using the standard default parameters or with the Multi-read correct –u parameter (“Multi-read Correct”), which assigns weighted FPKM values to loci with multi-mapping reads, based on an algorithm described previously [47].

FPKM values for individual HML-2 elements were used to calculate total HML-2 expression or packaging in the cells or virion by adding up the FPKM values from all HML-2 proviruses. From this number (“Total HML-2”), the percent abundance of each HML-2 provirus compared to the total value was calculated as (provirus FPKM)/(total HML-2 provirus FPKM) × 100 to illustrate the relative contribution of individual proviruses to total HML-2 expression or packaging in the cell or virion. Graphics were generated using Prism 6 (GraphPad software). Age estimates and open reading frames for proviruses were obtained from a previous publication [5] or by inputting sequence into the NCBI ORF Finder [48]. Heatmaps were created using RStudio (RStudio: Integrated development environment for R, version 0.98.1060). FPKM values for the heatmap were log-normalized using Decostand in RStudio Vegan and plotted using RStudio Pheatmap.

Alignments were visualized using the Integrative Genomics Viewer IGV v2.3.36 [49] and by using a custom track on the UCSC Genome Browser [50].

### 2.5. MiSeq In-Silico Simulation

Simulated MiSeq 250 base PE reads were generated from the faux HML-2 genome FASTA of 93 HML-2 proviruses to 20X coverage using the next-generation sequencing simulator program ART vVanillaIceCream-03-11-2014 [51]. Simulated reads were aligned back to the HML-2 genome using TopHat2 and were either kept “Unfiltered” or filtered for “Unique Only” alignment. FPKMs for each provirus were calculated using Cufflinks for both sets of FPKM values as previously described. As all proviruses were equally represented in the simulation, the average FPKM value for proviruses in the “Unfiltered” data set was used as the comparator for the “Unique Only” data set in order to assess which proviruses were underrepresented.

### 2.6. Phylogenetic Analysis

Neighbor-joining phylogenetic trees were created using MEGA6 [52]. Alignment of proviral or LTR sequence was performed using MUSCLE, an alignment tool native to the MEGA6 program. Neighbor-joining trees were constructed using the pairwise deletion option so that all available sites were used for comparison. The bootstrap values for the produced trees were the result of 1000 replicate tests. Distance was calculated using the p-distance method and the branch lengths correspond to the number of base differences per site.

### 2.7. LTR Amplification and Cloning

HML-2 proviral sequences were obtained from the hg19 build of the human genome using RepeatMasker in the UCSC Table Browser [53]. Primers flanking the 5’ LTR of each provirus were made using the Primer3 program [54] with restriction enzyme sites added to the 5’ ends of both the forward and reverse primers (Table S1). Primers used to create LTR truncation constructs are also reported (Table S2). Genomic DNA from Tera-1 cells was extracted with the DNeasy Blood & Tissue Kit (Qiagen, Cat# 69504) and used as a template for PCR amplification of the LTRs with *Taq* DNA polymerase (Invitrogen, Cat# 10342-020). The LTRs were directly cloned in sense orientation into the multiple cloning region of the pGL4.17[*luc2*/Neo] promoter-less firefly luciferase vector (Promega, Madison, WI, USA, Cat. #E6721). Reporter constructs were screened for mutations through sequencing before transfection.

### 2.8. Transfection and Dual-Luciferase Assay

Tera-1 cells were seeded at 1 × 10^5^ cell/well in a 24-well plate for transfection. The pGL4 firefly luciferase vector, containing the 5’ LTR of interest, was co-transfected alongside a pRL-SV40 internal control *Renilla* luciferase vector (Promega, Cat# E2231) at a 30:1 ratio, as recommended by the manufacturer’s protocol. Non-transfected Tera-1 cells were used as a control to account for any background signal associated with the assay. Transfections were carried out using Opti-MEM reduced-serum media (Life Technologies, Cat# 31985-070) and Lipofectamine 2000 (Life Technologies, Cat# 11668-019) according to the manufacturer’s protocol. The transfected cells were incubated for 48 h before lysis and assayed using the dual-luciferase assay system (Promega, Cat. #E1910). Luminescence was measured as relative light units (RLU) on a BioTek Synergy HT plate reader using Gen5 data analysis software (v2.03). The firefly luciferase signal was normalized against that of the *Renilla* luciferase signal to determine the relative promoter activity of each 5’ LTR.

## 3. Results

### 3.1. RNASeq Methodology to Determine HML-2 Provirus Expression

HML-2 proviruses are the most recently integrated ERVs in the human genome and have retained substantial coding potential [5]. In fact, the teratocarcinoma cell line Tera-1 expresses HML-2 RNA and protein and is capable of producing virions, though none have been found to be infectious [14]. The biology of this cell line is largely unknown, but it has been shown to primarily express HML-2 RNA originating from the provirus at chromosome 22q11.21 and other evolutionarily young integrations [13], and its virions appear to be immature and lacking Env glycoprotein [12].

**Figure 1:**
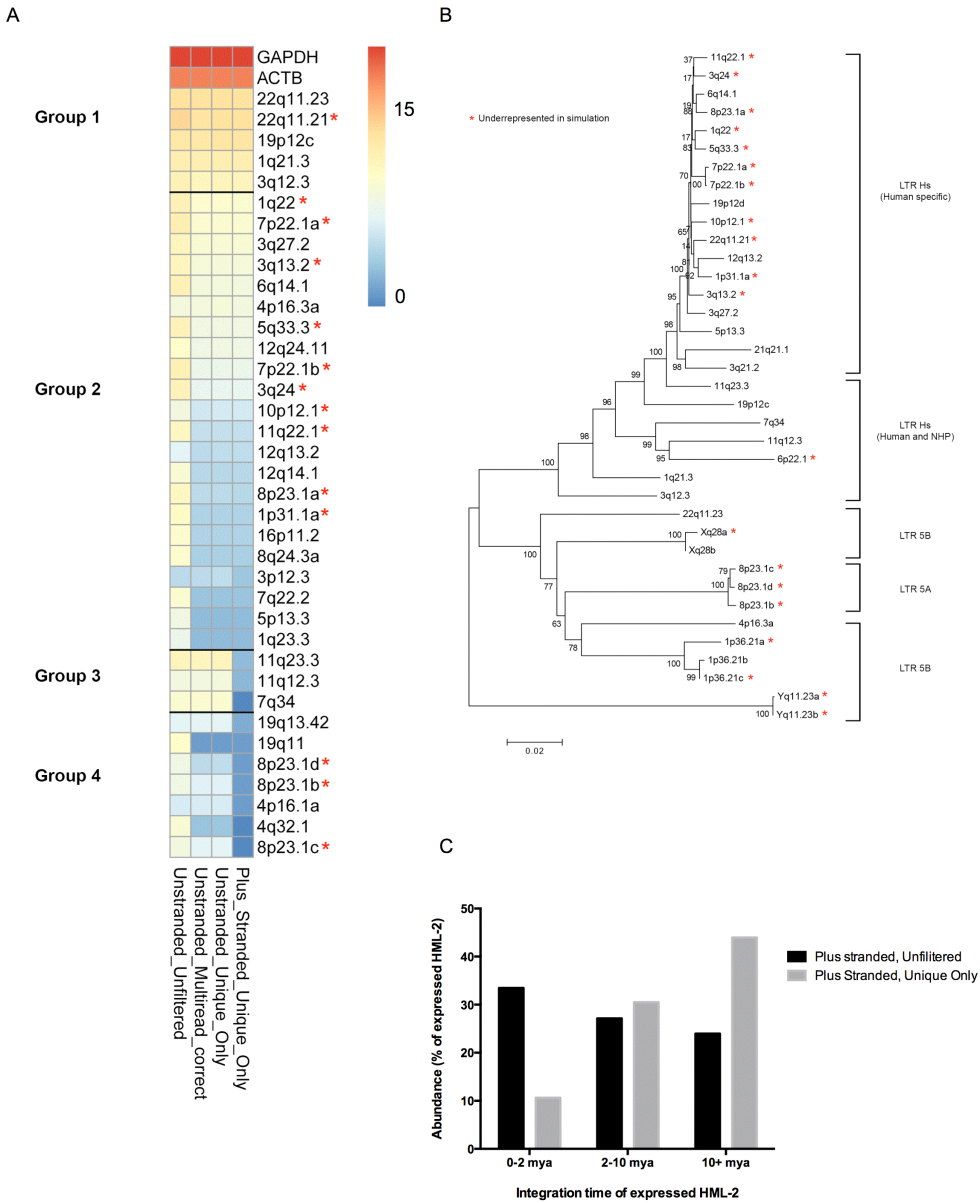
RNASeq analysis of HML-2 expression in Tera-1 cells. (**A**) RNASeq reads derived from Tera-1 cellular RNA were aligned to the hg19 build of the human genome, using either a stranded (“Plus Stranded”) or unstranded (“Unstranded”) alignment. Aligned reads were either kept in full (“Unfiltered”), or were filtered based on mapping quality scores to only retain reads that uniquely aligned to one map location (“Unique Only”). The fragments per kilobase per million mapped reads (FPKM) values representing relative expression in Tera-1 cells were determined either with a multi-read correct parameter (“Multi-read Correct”) that proportionally allocates multi-reads to mapping locations, or without this parameter. FPKM values for selected HML-2 proviruses and the cellular genes GAPDH and β-actin (ACTB) across the analyses were log-normalized and used for heatmap generation to demonstrate the effects of the different analyses on expression levels. Proviruses and gene loci are divided into four groups according to their relative values following the different analyses: stable (Group 1); decrease after Unique Only (Group 2); decrease after Plus stranded alignment (Group 3); and decrease after Unique Only and Plus stranded analysis (Group 4). Log-normalized FPKM is shown by the colors from high (red) to low (blue), as indicated in the chart to the right. The (*) symbols refer to proviruses predicted to be underrepresented by 15% or more based on an *in silico* simulation. (**B**) A neighbor-joining tree of the underrepresented proviruses was created using the full provirus sequence. The p-distance method was used and bootstrap values are indicated as percent of 1000 replicates. (**C**) The abundance of transcripts after the Plus stranded, Unfiltered and the Plus Stranded, Unique Only analyses are plotted against estimated times of integration to show the effect of the Unique Only analysis on recently integrated proviruses. The 0–2 mya group includes human specific integrations with high sequence similarity predicted to be underrepresented in the Unique Only RNASeq *in silico* simulation. The relative abundance in Tera-1 cells was calculated for each provirus based on (provirus FPKM)/(total HML-2 provirus FPKM) × 100. Elements without 5’ or 3’ LTRs were unsuitable for age estimation and are not included.

To discern more detail about the provirus expression profile of this unique virion-producing cell line, we applied RNASeq methodology to capture HML-2 proviral transcription. RNASeq provides a high-throughput approach to determine HML-2 expression in the context of other cellular genes and bypasses PCR primer bias in detecting HML-2 proviruses, as primers may not be able to detect older integrations due to sequence divergence. A predicted complication in applying this approach to HML-2 transcription profiling is caused by the high sequence similarity between recently integrated HML-2 proviruses (Figure 1B). Reads originating from highly similar or conserved areas could potentially align to multiple proviruses and interfere with RNASeq read alignment to a unique mapping location. Therefore, the effect of different RNASeq analysis methods on provirus representation was considered at each step.

Two RNASeq libraries were prepared for RNASeq analysis, with one constructed from Tera-1 cellular RNA and the other from Tera-1 virion RNA. Produced RNASeq reads were clipped of poor quality bases, adaptor sequences and reads shorter than 100 bases prior to alignment using TopHat2 [44].

Expression of some polymorphic proviruses may not be captured by alignment to the human reference genome because they were not present in the individual(s) contributing genomic sequence or were missed in genome assembly. Due to this anticipated issue, the reads were analyzed by TopHat2 alignment to the hg19 build of the human genome as well as to an HML-2 reference genome containing the sequences of 943 solo LTRs, 93 proviruses and a prototype SINE-R element, a type of retrotransposon comprised of HERV-K LTR and *env* sequence [55]. Of the 93 proviruses included in the HML-2 reference genome, four are present as solo LTRs in hg19 and two are present as pre-integration sites (Table S3). In the HML-2 reference genome, each element was listed as an independent sequence, thus functioning as a catalogue of 1037 HML-2 “chromosomes” during alignment.

Around half of all reads (~47%) aligned to HML-2 proviruses had multiple alignments, referred to as “multi-reads.” The true placement of a multi-read is in question since they may be misaligned to a closely related location and provide false signal for a provirus or solo LTR, leading to an inexact provirus transcription profile. Therefore, to circumvent this problem, only uniquely mapped reads were preserved, with the reasoning that a truly expressed provirus will also produce reads with unique sequence in addition to more conserved regions that will multi-map. Data were either kept in full (referred to as “Unfiltered”) or filtered for uniquely aligned reads (referred to as “Unique Only”).

Gene expression data from Unfiltered and Unique Only reads were used as input to calculate gene length normalized RNASeq expression values called FPKM (fragments per kilobase per million mapped reads) using the Cufflinks software package [46]. Cufflinks can assign multi-reads proportionally to multiple mapping locations based on abundance estimations for each mapping location. Reads were either analyzed in this manner (referred to as “Multi-read correct”) or were analyzed in a default manner where multi-reads are assigned to multiple mapping locations in a uniform manner (e.g., if a read maps to five locations, each location is assigned 20% of a read). Another parameter used in FPKM calculation is one that indicates whether transcription is occurring in the sense orientation for a particular locus. For the cell library, which was stranded, this parameter was used to properly estimate transcript abundance (referred to as “Plus stranded” if performed, and “Unstranded” if not).

A comparison of analytical methodologies of the RNASeq data is shown in a heatmap representation in Figure 1A, with yellow to red marking the highest expressed loci identified by the various approaches. All discussed proviruses are listed in Table 1 with their known aliases and genomic position. In the Unstranded, Unfiltered analysis, many proviruses are noted as expressed. However, in the Unique Only and Multi-read correct analyses, which consider only uniquely aligned reads or reads probabilistically assigned to loci, a dramatic drop off occurs in the FPKM of several of these “expressed” proviruses. Based on this analysis, we only considered proviruses remaining after either Unique Only filtering or Multi-read correction as being reliably expressed in Tera-1 cells and not misaligned to closely related loci. For example, the provirus 8p23.1a (K115; chr8: 7355397-7364859) is not present in Tera-1 cells [13]. Prior to filtering, this provirus was incorrectly assigned 2.5% of all HML-2 reads; after filtering, its expression level dropped to the background value of 0.07%. Of note, the Unique Only and Multi-read correct analyses gave virtually identical results in terms of assigning FPKM to specific proviruses for the Tera-1 data set. We additionally analyzed the effect of applying the strandedness option to FPKM assignment, thus considering only reads aligned in the sense orientation of the provirus. Unexpectedly, we found that a number of proviral loci were negatively affected by this distinction. These proviruses are displayed on the heatmap as becoming blue in the final column and include 7q34, 11q12.3 and 11q23.3. Without considering the strandedness of the read, these proviruses would not have been identified as products of antisense transcription, most likely due to neighboring transcription units. Specifically, in the case of the 7q34 provirus, reads that align to this locus appear to be the product of read-through transcription from the neighboring highly transcribed gene *SSBP1*. For the 11q12.3 provirus, which resides in an intron of the gene *ASRGL1*, aligned reads appear to be result of pre-mRNA present in the total RNA used for library preparation. Finally, at the proviral locus on 11q23.3, aligned reads appear to originate in a HERV-H element located just upstream of the proviral 3' LTR, though there did appear to be an increase in aligned reads throughout the provirus. The ability of proviral 3’ LTRs to drive antisense transcription was not tested in this analysis, though this appears to be most plausible for 11q23.3.

**Table 1:** Names and locations for discussed HML-2 proviruses.

Provirus	Alias	Chromosomal Location (hg19)
**1p31.1a**	K4, K116, ERV-K1	chr1: 75842771-75849143
**1p36.21a**	N/A	chr1: 12840260-12846364
**1p36.21b**	K(OLDAL023753), K6, K76	chr1: 13458305-13467826
**1p36.21c**	K6, K76	chr1: 13678850-13688242
**1q21.3**	N/A	chr1: 150605284-150608361
**1q22**	K102, K(C1b), K50a, ERVK-7	chr1: 155596457-155605636
**1q23.3**	K110, K18, 1 (+) K(C1a), ERVK-18	chr1: 160660575-160669806
**3p12.3**	N/A	chr3: 75600465-75609150
**3q12.3**	K(II), ERVK-5	chr3: 101410737-101419859
**3q13.2**	K106, K(C3), K68, ERVK-3	chr3: 112743123-112752282
**3q21.2**	K(I), ERVK-4	chr3: 125609302-125618416
**3q24**	ERVK-13	chr3: 148281477-148285396
**3q27.2**	K50b, K117, 3 (-) ERVK-11	chr3: 185280336-185289515
**4p16.1a**	K17b	chr4: 9123515-9133075
**4p16.3a**	N/A	chr4: 234989-239459
**4q32.1**	N/A	chr4: 161579938-161582360
**5p13.3**	K104, K50d	chr5: 30487114-30496205
**5q33.3**	K107/K10, K(C5), ERVK-10	chr5: 156084717-156093896
**6p22.1**	K(OLDAL121932), K69, K20	chr6: 28650367-28660735
**6q14.1**	K109, K(C6), ERVK-9	chr6: 78427019-78436083
**7p22.1a**	K108L, K(HML.2-HOM), K(C7), ERVK-6	chr7: 4622057-4631528
**7p22.1b**	K108R, ERVK-6	chr7: 4630561-4640031
**7q22.2**	ERVK-14	chr7: 104388369-104393266
**7q34**	K(OLDAC004979), ERVK-15	chr7: 141450926-141455903
**8p23.1a**	K115, ERVK-8	chr8: 7355397-7364859
**8p23.1b**	K27	chr8: 8054700-8064221
**8p23.1c**	N/A	chr8: 12073970-12083497
**8p23.1d**	KOLD130352	chr8: 12316492-12326007
**8q24.3a**	N/A	chr8: 140472149-140475236
**10p12.1**	K103, K(C10)	chr10: 27182399-27183380
**11q12.3**	K(OLDAC004127)	chr11: 62135963-62150563
**11q22.1**	K(C11c), K36, K118, ERVK-25	chr11: 101565794-101575259
**11q23.3**	K(C11b), K37, ERVK-20	chr11: 118591724-118600883
**12q13.2**	N/A	chr12: 55727215-55728183
**12q14.1**	K(C12), K41, K119, ERVK-21	chr12: 58721242-58730698
**12q24.11**	N/A	chr12: 111007843-111009325
**16p11.2**	N/A	chr16: 34231474-34234142
**19p12a**	K52	chr19: 20387400-20397512
**19p12b**	K113	chr19: 21841536-21841542 (empty site)
**19p12c**	K51	chr19: 22757824-22764561
**19p12d**	N/A	chr19: 22414379-22414382 (empty site)
**19q11**	K(C19), ERVK-19	chr19: 28128498-28137361
**19q13.42**	LTR13	chr19: 53862348-53868044
**21q21.1**	K60, ERVK-23	chr21: 19933916-19941962
**22q11.21**	K101, K(C22), ERVK-24	chr21: 18926187-18935307
**22q11.23**	K(OLDAP000345), KOLD345	chr21: 23879930-23890615
**Xq28a**	K63	chrX: 153817163-153819562
**Xq28b**	K63	chrX: 153836675-153844015
**Yq11.23a**	N/A	chrY: 26397837-26401035
**Yq11.23b**	N/A	chrY: 27561402-27564601

The exclusion of reads that align to multiple map locations may create a reporting bias, in which highly similar proviruses could be underrepresented due to a paucity of uniquely aligned reads. To determine the effect of this approach on HML-2 expression analysis, an *in silico* simulation of the RNASeq Unique Only analysis was performed as described in Materials and Methods. Based on this simulation, we found that mostly recently integrated proviruses and duplications appeared to be underrepresented after filtering for unique reads. The proviruses that were negatively affected by >15% (range: 17%–86%) are shown on the neighbor-joining phylogenetic tree in Figure 1B. Three main categories of HML-2 proviruses, 5A, 5B, and Hs, have been recognized based on their LTR phylogenies. LTR 5B proviruses are basal to both LTR 5A and LTR Hs proviruses, the latter of which include the more recent, mostly human-specific, HML-2 integrations [5]. Many of the underrepresented loci include the most recent LTR Hs integrations, which have accumulated fewer mutations since their last common ancestors than those resident in the genome for longer periods of time (Figure 1B). In addition, proviruses that are known to have arisen by duplication post-integration, including the LTR Hs proviruses on 7p22.1, the LTR 5B proviruses on 1p36.21, Xq28 and Yq11.23, and the LTR 5A proviruses on 8p23.1, are also represented in the RNASeq simulation less frequently than expected (Figure 1B). Curiously, the LTR Hs provirus 6p22.1, which is not human specific, was also underrepresented in the simulation. However, all proviruses were detected in the simulation. Therefore, although the true abundance of affected proviruses may be underrepresented, their expression will, nevertheless, be captured in the analysis. All proviruses that appeared to be affected in this way are shown with a red asterisk in Figure 1A,B to denote their potential underrepresentation. Of interest, the two LTR Hs proviruses that cluster tightly and exhibit long branch length, 3q21.2 and 21q21.1, were shown to have been hypermutated by APOBEC3G [56].

To depict how the Unique Only analysis affected provirus representation in Tera-1 cells, Figure 1C shows how the estimated age of integration for expressed proviruses changed between an Unfiltered, Plus stranded alignment and the Unique Only, Plus stranded alignment. Recently integrated proviruses are still represented in analysis, however they are 2/3 less abundant, leading to a perceived overrepresentation of older elements.

### 3.2. Expression and Packaging of HML-2 Proviruses in Tera-1 Cells and Virions

**Figure 2:**
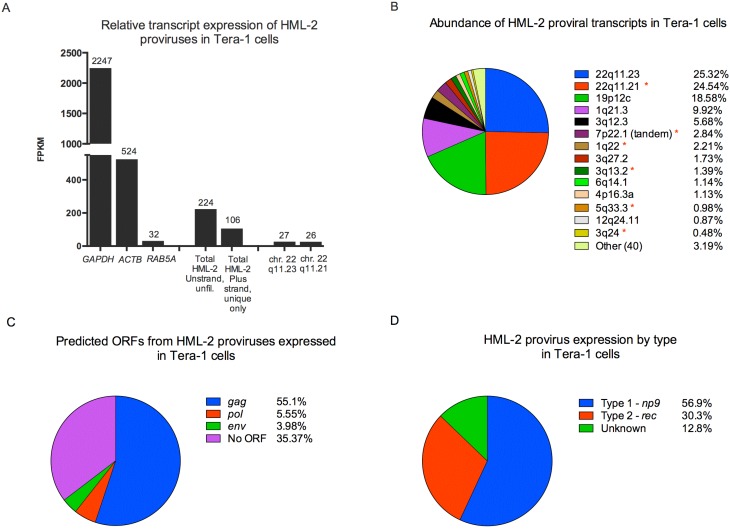

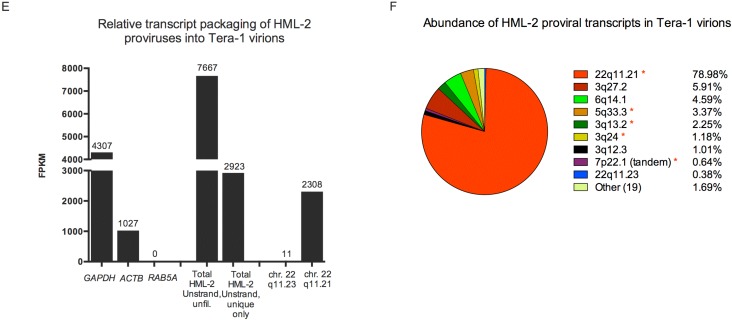
HML-2 expression in Tera-1 cells and virions. (**A**,**B**) RNASeq reads originating from Tera-1 cells were aligned to the hg19 build of the human genome and analyzed using the Plus stranded, Unique Only analysis, except as indicated. (**E**–**F**) RNASeq reads originating from Tera-1 virions were aligned to the hg19 build of the human genome and analyzed using the Unstranded, Unique Only analysis, except as indicated, due to the input library not being stranded. (**A**, **E**) Relative transcript expression values (FPKM) for cellular genes, total HML-2 and the most abundantly expressed or packaged HML-2 transcripts are plotted for Tera-1 cells (**A**) and Tera-1 virions (**E**). (**B**,**F**) Abundance of transcripts for each provirus in Tera-1 cells (**B**) and virions (**F**) is plotted according to (provirus FPKM)/(total HML-2 FPKM) × 100. Proviruses with (*) were predicted to be underrepresented by the *in silico* analysis, as used in Figure 1. (**C**) Open reading frames for *gag*, *pol* and *env* were determined for proviruses making up 96.81% of all HML-2 reads shown in Figure 2B. If a provirus had the potential to express open reading frame(s) (ORF(s)), the abundance of the provirus in the cell was allocated to each ORF, as this represents the maximum probability of that ORF being expressed. Splicing was not considered for this analysis. (**D**) Type 1/2 status was determined for HML-2 proviruses making up 96.81% of all HML-2 reads, listed in Figure 2B. Unknown indicates that the entire *pol*-*env* boundary region was not present in the provirus, preventing identification of provirus type.

Using the Unique Only, Plus stranded approach, HML-2 transcription in Tera-1 cells was quantified relative to the cellular genes *GAPDH*, *ACTB* and *RAB5A* (Figure 2A). The analysis removed roughly half of all HML-2 reads present in the unfiltered alignment, yet total expression of this group was still readily quantifiable, at ~1/200th the level of the metabolic gene *GAPDH* and ~1/5th the level of the cytoskeletal gene *ACTB* (β-actin). The top two expressed HML-2 proviruses, the LTR 5B provirus at 22q11.23 (see later for more on this provirus) and the LTR Hs provirus at 22q11.21 (Figure 2A,B), were each detected at a level comparable to that of the cellular gene *RAB5A*, which encodes a protein localized on early endosomes, and together made up roughly half of all the HML-2 reads generated from Tera-1 cells.

LTR Hs type proviruses were the most commonly expressed proviruses in Tera-1 cells (12 out of the top 14), and included 7 human specific integrations that were likely to be underrepresented (indicated with red asterisks in Figure 1A,B and Figure 2B). The tandem duplicated LTR Hs proviruses on chromosome 7p22.1 [5] were considered together since they are nearly identical in sequence and reads could have originated from either of them. Interestingly, two ancient LTR 5B proviruses, on chromosomes 22q11.23 and 4p16.3a, were also expressed in the cells but no LTR 5A proviruses were detected at >0.2% abundance.

The open reading frames (ORFs) for the genes *gag*, *pro*, *pol*, and *env* vary among the HML-2 proviruses. To assess the contribution of the identified proviruses to virion production, we calculated the relative numbers of transcripts belonging to proviruses capable of potentially expressing full-length gene products. We found that most (55.1%) expressed HML-2 sequence was capable of encoding *gag*, largely from the *gag* ORFs present on proviruses 22q11.23 and 22q11.21 (Figure 2C). Of interest, the Gag protein encoded by 22q11.21 appears to be full length (666 amino acids) but the one encoded by 22q11.23, which is a more ancient provirus, is predicted to be truncated by 43 amino acids at the C-terminus. The *pol* (5.6%) and *env* (3.9%) ORFs were much less well represented, and a significant amount (35.4%) of the expressed HML-2 sequence was derived from proviruses that lack coding capability altogether (Figure 2C). The majority of HML-2 proviruses expressed were Type 1 (Figure 2D), which is typified by a 292-bp deletion at the *pol*-*env* boundary, resulting in a non-functional Env, and encodes the accessory gene *np9* [57]. Type 2 proviruses, which made up 30% of expressed HML-2 proviruses, retain full sequence at the *pol*-*env* boundary and encode the accessory gene *rec* [58]. The detection of doubly spliced transcripts for *np9*, *rec* and the non-coding transcript *hel* [20,58] is detailed for individual proviruses in Table S4.

In reads generated from Tera-1 virions, HML-2 sequences were more frequently represented compared to their detection in the cells, as expected, exhibiting >25-fold increase in FPKM (Figure 2E). Virions also appeared to non-specifically package the highly expressed cellular mRNAs from *GAPDH* and *ACTB*, which were increased about two-fold in FPKM from their levels in cells, but not *RAB5A*, which was not detected in the virions (Figure 2E). Relative to *GAPDH*, HML-2 representation increased >100-fold in virions, and in comparison to *ACTB*, ~10-fold (Figure 2E).

The virion reads aligned primarily to the type 1 provirus on chromosome 22q11.21, making up ~79% of all HML-2 reads (Figure 2E–F). This observation is in agreement with a previous publication assessing the origins of packaged HML-2 RNA from Tera-1 virions [13]. In virions, over 90% of packaged genomes originated from Env-defective Type 1 proviruses, mainly due to the abundance of the 22q11.21 transcripts. The top 6 packaged proviruses are all members of the human specific LTR Hs group, a major distinction from the proviruses expressed in Tera-1 cells, which included LTR Hs proviruses that were not human specific as well as an older LTR 5B proviruses (Figure 2B).

### 3.3. Human Specific LTR Hs Proviruses were Enriched in HML-2 Virions

**Figure 3:**
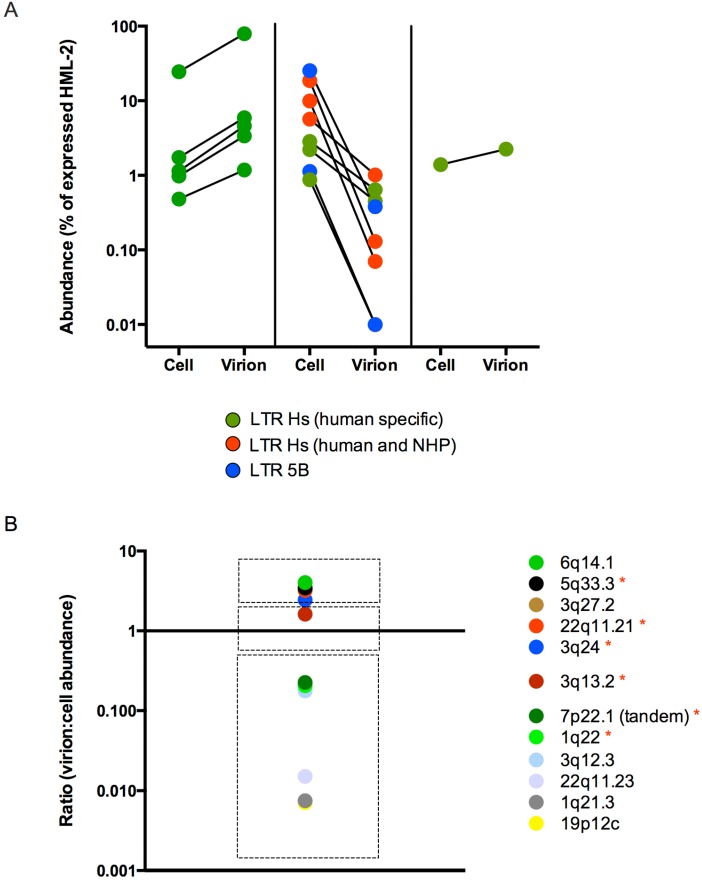
HML-2 packaging shows preference for recently integrated proviruses. (**A**) The abundance of proviruses expressed in the cell and packaged into virions was calculated as described in Figure 2. These values were plotted side-by-side to show an increased abundance (panel 1, left), decreased abundance (panel 2, middle) or similar abundance (panel 3, right) for proviruses packaged in virions as compared to their expression in the cell. Long terminal repeat (LTR) types of proviruses detected are indicated, with LTR Hs (human specific) in green, LTR Hs (in humans and non-human primates) in red and LTR 5B in blue. Two proviruses (12q24.11 and 4p16.3a) that were not detected in virions were plotted at 0.01% in panel 2. (**B**) The identities of the proviruses and the ratios of their virion to cell abundance are shown. Proviruses with (*) were predicted to be underrepresented by the *in silico* analysis (Figure 1).

HML-2 transcripts packaged in Tera-1 virions were more abundant, less abundant or present in roughly equal proportion to their expression in cells (Figure 3A), and this pattern reflected the relative time of their integration. The transcripts with the highest increase in abundance in virions were all derived from recently integrated human specific LTR Hs proviruses, some of which were predicted to be underrepresented in the analysis (Figure 1B), noted with red asterisks as before (Figure 3B), whereas those that decreased in abundance mostly originated from either older LTR Hs or LTR 5B proviruses. For example, transcripts from the LTR 5B provirus at 22q11.23 made up 25.32% of all cellular HML-2 reads, but its abundance in the virions it was 0.38%, a 66-fold decrease (Figure 3B). Transcripts from the LTR 5B provirus at chromosome 4p16.3a, expressed in cells at about 1%, were not even detected in the virions. Other transcripts with major decreases, on chromosomes 1q21.3, 3q12.3 and 19p12, were derived from older LTR Hs integrations.

### 3.4. HML-2 Proviruses are Transcribed through a Variety of Mechanisms

**Figure 4:**
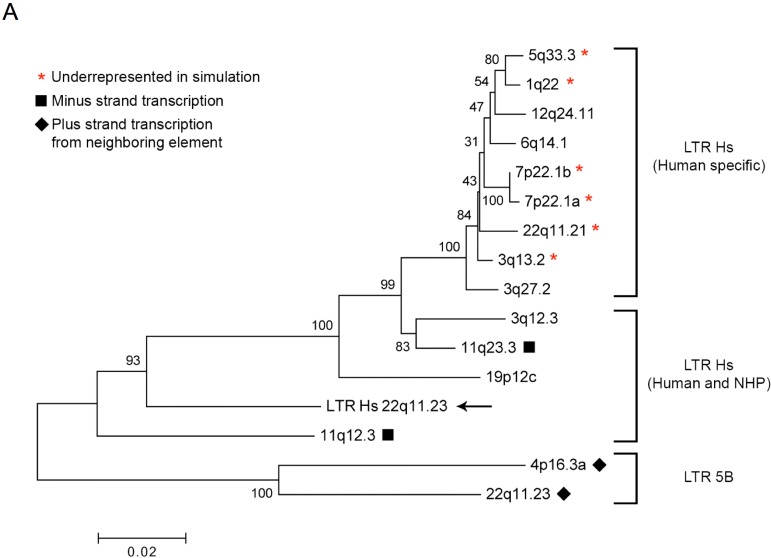

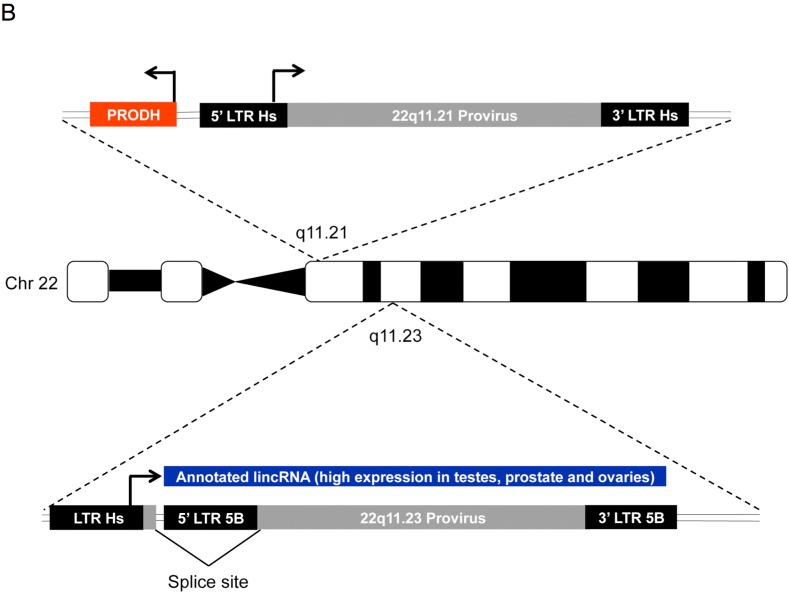
Transcription of HML-2 proviruses is driven by the native LTR or a nearby element. (**A**) Neighbor-joining tree of the 5’ LTR sequences of the HML-2 proviruses expressed in Tera-1 cells. The p-distance method was used to calculate distance and bootstrap values are indicated (1000 replicates). Proviruses with (*) were predicted to be underrepresented by the *in silico* analysis, as in Figure 1. Solid squares (∎) indicate those proviruses (11q23.3 and 11q12.3) with minus strand transcription. Solid diamonds (♦) indicate those proviruses (4p16.3a and 22q11.23) with plus strand transcription, but which appear to originate from a neighboring transcription unit and not the corresponding 5’ LTR. (**B**) A cartoon of two proviruses located on chromosome 22 and their method of transcription. Provirus 22q11.21 (LTR Hs, FPKM = 26.11) is located 2.1 kb downstream from the expressed gene *PRODH* (Proline Dehydrogenase (oxidase) 1, FPKM = 11.53) but in the opposite transcriptional orientation. The 5’ LTR of 22q11.21 appears to drive proviral transcription in Tera-1 cells. Provirus 22q11.23 (FPKM = 26.94) appears to be transcribed solely through the use of an LTR Hs (FPKM = 0.31) located 551 bp upstream from the provirus. This transcript coincides with an annotated lincRNA (large intergenic non-coding RNA) [59]. See supplemental Figures S3 and S4 for more detail. Cartoon is not drawn to scale.

To determine the relatedness of the expressed proviruses shown in Figure 2B, the relationship of their 5’ LTRs was visualized using a neighbor-joining tree [52] (Figure 4A). As expected, the recently integrated (human-specific) LTR Hs proviruses clustered very closely and for the most part could not be definitively assigned to branches due to their similarity, as seen with the low bootstrap support values generated (Figure 4A). However, the relationship of the older LTR Hs elements and LTR 5B elements could be ascertained from the tree and was clearly distinct from the recent LTR Hs integrations. The association of divergent LTR types with transcribed proviruses in the Tera-1 cells implies either that the promoter elements of these distinct LTRs were all functional, or that there are alternative ways (*i.e.*, 5’ LTR independent) in which some of the proviruses were transcribed.

Visualization of HML-2 reads aligned to their map locations using the UCSC Genome Browser [50] or the Integrative Genomics Viewer [49] can inform whether transcription of a provirus is driven by its 5’ LTR. That is, 5’ LTR driven proviral transcription should be confined to the provirus itself, whereas transcription caused by read-through from a neighboring transcription unit results in reads aligning to the provirus as well as flanking sequence intermediate to the transcriptional start and/or end. Transcription driven from a neighboring element may also result in minus strand reads if the provirus and element are in opposite transcriptional orientation, a phenomenon relevant to LTR Hs proviruses 7q34, 11q12.3 and 11q23.3 (Figure 1A and Figure 4A, solid squares).

In contrast, transcription of LTR 5B proviruses 4p16.3a and 22q11.23 (Figure 4A, diamonds) appears to be driven by sequences other than the corresponding 5’ LTR. Provirus 4p16.3a (FPKM = 1.19) resides in an intron for the expressed gene *ZNF876P* (FPKM = 9.45) and reads align evenly to the pre-mRNA intronic sequence, including the provirus. This result implies that the provirus is not being specifically transcribed; rather, it is preserved in an incompletely removed intron. Remarkably, visualization of the highly expressed LTR 5B 22q11.23 provirus (FPKM = 26.94) revealed a fragmentary LTR Hs element (FPKM = 0.31) 551 bp upstream, which appeared to be the start site for some fraction of 22q11.23 proviral transcription (Figure 4B, Figure S3). Transcription appeared to start midway through the R region of the upstream LTR Hs at position 826. Splicing of the transcript occurred at position 1074 (*gag* leader) of the LTR Hs element into position 1018 (*gag* leader) of the LTR 5B provirus and followed the GU-AG rule (Figure 4B). This spliced transcript has been annotated as a lincRNA (TCONS_l2_00017644; (Cabili, 2011 #11601)), though its function is unknown. Since reads aligned to the 22q11.23 proviral 5’ LTR 5B could be indicative of promoter activity, the amount of transcription originating from the upstream 5’ LTR Hs could not be accurately estimated. Of note, the FPKM value for the upstream LTR Hs appeared to be artificially low since the reads primarily aligned only to a small region at the end of the element. The relationship of the 22q11.23 LTR Hs sequence to that of other expressed LTR Hs sequences is shown in Figure 4A (black arrow).

The read-through transcription that appears to be driving expression of the LTR 5B proviruses can be contrasted with the clearly 5’ LTR driven transcription of the top expressed LTR Hs provirus, 22q11.21 (FPKM = 26.1) (Figure 2A,B). This provirus is integrated 2.1kb downstream from the transcriptional start of the expressed cellular gene *PRODH* (FPKM = 11.53). Their transcriptional orientations are divergent (Figure 4B, Figure S4), although their expression has been reported to be linked [60]. Due to the sequence similarity of this provirus with other recently integrated LTR Hs proviruses, some internal and LTR regions do not show coverage after the Unique Only filter is applied (Figure S4). The transcriptional start for this provirus appears to occur around and after position 780 on the 5’ LTR, near or at the expected site at the U3-R border at 793. In support of the role of the 5’ LTR in driving proviral transcription, there are only a few reads aligned to the flanking region upstream of the provirus, indicating that upstream elements do not contribute to provirus transcription.

### 3.5. 5’ LTR Activities of Expressed HML-2 Proviruses Corroborate RNASeq Findings

**Figure 5:**
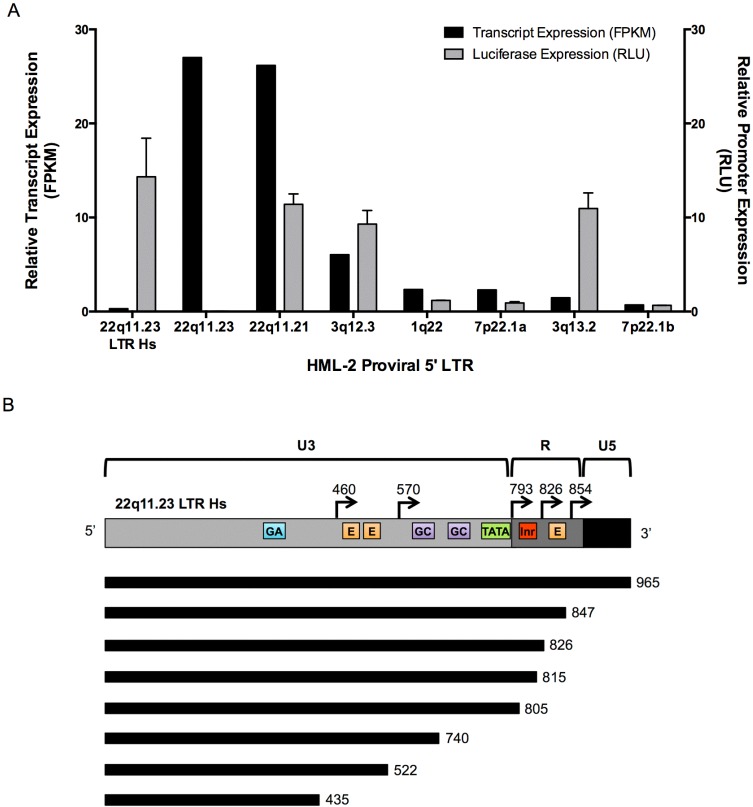

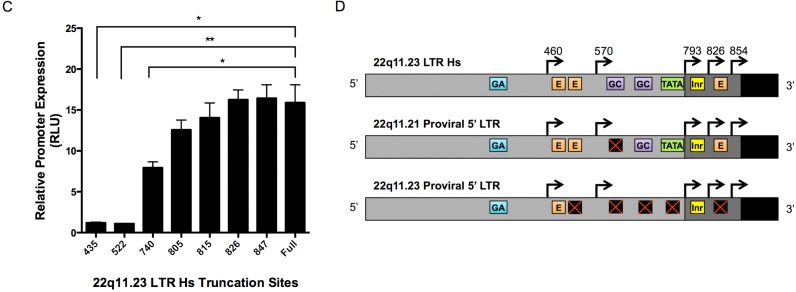
HML-2 Promoter Expression in Tera-1 Cells. (**A**) Comparison of the relative transcript expression level (FPKM; black) for a provirus and its corresponding relative luciferase expression level in Tera-1 cells transfected with a vector containing a luciferase reporter gene downstream of the indicated proviral 5’ LTR. LTR activity is expressed as relative light units (RLU; gray) normalized to a control construct with a *Renilla* luciferase gene driven by an SV40 promoter. The relative promoter activities of the LTR Hs located 551 bp upstream from the 22q11.23 provirus, the 5’ LTR 5B of the 22q11.23 provirus and the 5’ LTR Hs of six other expressed proviruses in Tera-1 cells are shown. (**B**) Schematic of the 22q11.23 LTR Hs, showing the U3, R and U5 regions. Predicted transcriptional start sites are indicated with black arrows and nucleotide position. Colored boxes indicate previously described promoter element motifs [62,63,64]. Lines below the LTR diagram indicate the regions included in each truncated LTR construct, and numbers to the right of each line indicate the nucleotide position at which the LTR was truncated. GA, GA rich motif (nt 379–386, sequence GGGAAGGG); E, enhancer box (nt 465–476, sequence TTGCAGTTGAGA; nt 485–496, sequence AGGCATCTGTCT; nt 832–843, sequence CTCCATATGCTG); GC, GC rich motif nt 759–763, (sequence CCCCC; nt 602–606, sequence GGCGG); TATA, TATA box (nt 790–797, sequence AATAAATA); Inr, initiator element (nt 807–812, sequence CTCAGA). Cartoon is not drawn to scale. (**C**) Relative promoter expression levels of truncated 22q11.23 LTR Hs constructs in Tera-1 cells (Kruskal-Wallis, * *p* < 0.05, ** *p* < 0.01). All luciferase experiments were conducted in triplicate and are shown as mean ± standard deviation. (**D**) Schematic of promoter motifs found in the 22q11.21 provirus 5’ LTR Hs, the 22q11.23 LTR Hs and 22q11.23 provirus 5’ LTR 5B. Crossed out boxes indicate presence of a mutation in the motif as compared to the canonical sequence. Cartoon is not drawn to scale.

Retroviral 5’ LTRs generally possess all promoter elements necessary to drive the transcription of associated viral genes [3,61,62]. However other factors in cells, such as epigenetic effects and expression of nearby genes may also affect their transcription. To investigate the correlation of transcription and promoter activity, we cloned 5’ LTRs from seven expressed proviruses into luciferase constructs and assessed their function following transfection of Tera-1 cells. The LTR Hs upstream of the 22q11.23 provirus was similarly assayed for activity to address its role in driving expression of the ancient provirus in lieu of the proviral 5’ LTR 5B. The relative promoter activity for each assayed LTR was calculated as relative light units (RLU) normalized to that of a co-transfected control containing the SV40 promoter. Figure 5 shows the promoter activity of each LTR compared directly to the FPKM value of the originating provirus, as determined by the Unique Only, Plus stranded alignment detailed in Figure 2A,B.

As shown in Figure 5B, the 5’ LTRs from most expressed proviruses displayed relative promoter activities comparable to their associated relative FPKM. Most provirus promoter activity varied ±2.5-fold from the reported FPKM values. A major exception to this pattern was seen with the 22q11.23 provirus, whose LTR 5B was >500-fold less active relative to its FPKM (Figure 5A), while the activity of the upstream LTR Hs appeared to correlate with the FPKM of its transcripts. This result, taken together with the alignment data showing read-through transcription between the 22q11.23 LTR Hs and the downstream LTR 5B provirus (Figure S3), is consistent with the conclusion that the high expression of the 22q11.23 provirus in Tera-1 cells is due to the upstream LTR Hs, which is capable of high promoter activity. Another provirus whose promoter activity did not correspond well with its FPKM value was 3q13.2, which has predicted to be underrepresented in Figure 1. 3q13.2 displayed a 7.6-fold higher promoter activity level as compared to its FPKM, which may indicate that this provirus was underrepresented in the RNASeq analysis.

The canonical HML-2 LTR transcription start site is believed to be located at position 793 (Figure 5C) [62,63]. However, the RNASeq alignment showed 22q11.23 LTR Hs transcripts originating from further downstream, primarily at position 826 (Figure S3). Potentially, this LTR Hs can use an alternative start site to initiate transcription. To investigate this issue further, a series of truncated LTR Hs constructs containing varying promoter associated elements (Figure 5C) was transfected into Tera-1 cells and analyzed for activity as described for Figure 5A,B.

We observed only small decreases in activity after truncating the 3’ end of the LTR to position 805, an unexpected result, considering the RNASeq alignment data showing a TSS at 826 (Figure 5D and Figure S3). No significant drop in activity was seen until the LTR was truncated to position 740, resulting in removal of both a GC box and the TATA box (Figure 5C). In addition, truncations down to positions 522 and 460 ablated LTR activity almost entirely (Kruskal-Wallis, * *p* < 0.05, ** *p* < 0.01, Figure 5D). Thus, the ability of the LTR Hs to promote transcription is largely dependent on GC box and/or TATA box promoter elements in the luciferase assay and did not appear to mimic the TSS exhibited in its native genomic location, although we have not yet directly determined its site of transcriptional initiation.

The most active relatively recent LTRs in the Tera-1 cells, namely the 5’ LTR of the 22q11.21 provirus and the LTR Hs on 22q11.23, may have preserved promoter elements that allowed for their activity, in comparison to less active, older LTRs, like the 22q11.23 5’ LTR 5B. Accordingly, canonical E box, GC box and TATA box elements were found in the most active LTRs (Figure 5E). Conversely, the mostly inactive 22q11.23 LTR 5B displayed 1–3 nucleotide mutations for 5 out of the 8 promoter elements on its 5’ LTR (Figure 5E). Significantly, this LTR did not retain the canonical GC box or TATA box sequences, which were shown to be important for HML-2 LTR transcription in Figure 5D. Thus, HML-2 LTR activity in Tera-1 cells appears to be in part reliant on maintenance of canonical promoter motifs.

## 4. Discussion

HERV-K (HML-2) proviruses represent the most recently integrated proviruses in the human genome, some of which maintain ORFs for retroviral genes [5]. Although not expressed in most normal tissue, multiple studies have shown HML-2 transcription to be associated with several disease states, notably in cancers and HIV-1 infection [20,65,66]. Neither the mechanism nor the consequences of this expression are well understood. An impediment to investigating their possible role in disease has been the incomplete understanding of their expression patterns in human cells and, along the same lines, the availability of only small sample sizes of analyzed tissues [67,68]. In addition, the exact proviral loci and the details of their regulation that may be important in a disease context are not known. To address these issues, we applied RNASeq analysis to capture the HML-2 transcription profile of the teratocarcinoma cell line Tera-1 and to determine HML-2 packaging in the virions produced from this cell line.

Our approach to HML-2 profiling was to create total RNA Illumina MiSeq libraries from Tera-1 cells and virions, with a maximum sequencing length of 301 bases per read. By using RNASeq instead of PCR-cloning to characterize Tera-1 HML-2 transcription, an approach used previously [13], we were able to bypass the effects of PCR primer and cloning bias in provirus amplification, and thus achieve greater sensitivity in the breadth of proviruses identified as both expressed in cells and packaged into virions. In addition, by performing a total RNA analysis, the context of provirus expression was understood. As reported, the HML-2 transcription profile in Tera-1 cells included both recent and older proviruses as well as plus strand and minus strand transcription, results that would not have been captured by PCR-cloning. Furthermore, we discovered that the transcription of the most highly expressed LTR 5B provirus was in fact driven by an LTR Hs element upstream, while others appeared to be driven by their native LTRs or neighboring transcription units, exemplifying how RNASeq captures valuable contextual data about how HML-2 elements are expressed in specific cells.

Recently, two groups applied next-generation sequencing to address HML-2 expression in primary human PBMCs [38,39]. We consider our approach as combining positive attributes from both methods. The lower error rate in Illumina sequencing may offer an advantage over PacBio sequencing, in addition to bypassing the effects of RT and PCR primer bias in amplicon sequencing for identification of closely related proviruses [38]. However, the PacBio platform offers longer reads lengths that would be ideal for HML-2 identification. In comparison to the Illumina HiSeq method used previously [39], our approach similarly only considers uniquely mapped reads, but takes advantage of the 3× longer read lengths available through Illumina MiSeq platform, thus making unique identification of HML-2 proviruses more apparent. Furthermore, in our libraries, by keeping the RNA unsheared or by limiting fragmentation, we created a pool of longer library inserts that allows for improved provirus identification, especially in combination with paired-end sequencing, alignment and expression analysis as we performed. Lastly, in contrast to both previous methods, our RNASeq analysis fits within the well-established TopHat-Cufflinks pipeline used in multiple fields for transcriptome analysis, offering a streamlined approach to HML-2 expression profiling without the necessity of custom scripting or higher-level bioinformatics.

The high sequence similarity among the recently integrated HML-2 proviruses (Figure 1B, Figure 4A, and S2) was predicted to complicate RNASeq analysis. Reads generated from areas of high sequence similarity can cause the phenomenon of “multi-reads,” where the read will map to multiple locations in the reference genome. As a testament to the sequence similarity between proviruses, close to 50% of all reads that mapped to HML-2 proviruses were multi-reads in the Tera-1 cell RNASeq library, and ~60% in the Tera-1 virion RNASeq library (Figure 2A,C). This observation speaks to the high number of recently integrated HML-2 proviruses expressed in these samples; however, it causes substantial confusion in terms of assigning the read to a specific locus. Accurate locus-specific assignment is critical to understand the biological relevance of HML-2 expression. To circumvent this complication, we used a filter to consider only uniquely mapped reads for the transcription profile. Although the RNASeq *in silico* simulation (Figure 1B) showed that this approach underrepresented both human specific LTR Hs proviruses as well as known duplicated proviruses in the genome, importantly, it is still able to capture their expression, albeit at a lower level. As sequencing read lengths increase, the alignability of reads from these highly related loci will correspondingly increase and the effects of a conservative Unique Only alignment should not hamper detection of modestly expressed loci. An approach to maximize the utility of data generated from Illumina MiSeq is to custom prepare libraries so that the RNA input is not over sheared, which negatively limits the insert size available for sequencing, and also to enrich the library for longer inserts, which can be achieved using size selection during library preparation. These steps would circumvent the favored sequencing of shorter molecules during the sequencing reaction and give longer sequence for alignment in downstream analysis. In our analysis, we found that the relative transcript expression values (FPKM) of the most highly transcribed proviruses did not appear to be greatly affected by this Unique Only approach, although detection of lower transcribed proviruses was impaired (Figure 1A).

LTR activity assays (as performed in Figure 5) were used to ascertain whether the Unique Only approach did remove proviruses legitimately expressed at low levels (Figure S5). In this analysis, 5’ LTRs from three proviruses (1p31.1a, 11q22.1 and 12q13.2) that showed a decrease in relative expression after Unique Only analysis and were detected at lower than 0.5% of all HML-2 reads in the hg19 alignment were cloned and assayed for LTR activity. LTR activity was then compared back to either the Unfiltered FPKM or the Unique Only FPKM generated for the locus. LTR activities of the selected loci appear to relate to the Unique Only expression value, potentially extending this analysis approach even for poorly expressed loci. However, even though the Unique FPKM and LTR activity values look similar, the exact relationship of the proviral relative transcript expression value to its LTR activity is not established and cannot be interpreted definitively.

Filtering out multi-reads can lead to gaps in read coverage for transcripts from closely related proviruses. For the LTR Hs provirus at 22q11.21, almost full proviral coverage is seen when reads are Unfiltered (Figure S4A). However, after Unique Only selection, coverage is clearly limited to several unique portions of the genome (Figure S4B). Another way coverage of a provirus can be interrupted is due to polymorphisms in the donor sequence that are not present in the reference. For example, a region in the *pol* gene of provirus 22q11.21 does not have substantial read coverage in either the Unfiltered or Unique Only alignments (Figure S4A,B, red arrow). Depending on the provirus, regions missing reads could indicate that there are mutations in the donor sequence that causes them to misalign to other related proviruses, or potentially remain unaligned if too divergent. Through sequence analysis of the 22q11.21 provirus, we established the presence of four SNPs over ~200 bases in *pol* that overlapped the gap in read coverage. If gaps in coverage for a provirus in the unfiltered alignment are pervasive and do not correspond to the presence of SNPs in the donor sequence, the validity of read assignments to it could be called into question.

Another issue that arises in mapping HML-2 transcription is that not all known integrations are annotated in the hg19 build of the human genome. As mentioned previously, some HML-2 proviruses are insertionally polymorphic within the human population, others are found as solo LTRs in some individuals, and full-length proviruses in others. To ensure that we captured all known proviruses, an HML-2 reference “genome” was assembled containing 943 solo LTR and 93 proviral sequences, 6 of which were not present, or only partially present, in hg19 (Table S3). Thus, an alignment to the HML-2 reference genome was run in parallel to the hg19 alignment to validate hits. As seen in Figure S1A-D, the abundance values generated in the HML-2 reference alignment generally corroborated the proviruses found using hg19. A notable difference is that a provirus not present in hg19, referred to as 19p12d (empty site in hg19: 22414379-22414382, not K113), appeared to be expressed in Tera-1 cells.

Based on the LTR phylogeny (Figure 4A), the LTR Hs group of proviruses was much more highly represented than the older LTR 5A and 5B groups in the Tera-1 cell transcriptome. Furthermore, transfection assays using the various LTRs to drive expression of a luciferase reporter in Tera-1 cells showed levels of expression consistent with the relative transcript levels, at least for most of the LTR Hs proviruses (Figure 5). Consistent with this observation, the 5’ LTR 5B of the provirus at 22q11.23 had relatively low transcriptional activity compared to the upstream LTR Hs. This difference could be due to the absence of GC and TATA boxes, since deletion experiments showed that the region containing these elements was important for retaining transcriptional activity of the 22q11.23 LTR Hs in Tera-1 cells (Figure 5E), although the individual contributions of each was not discerned from the assays. Potentially, the GC boxes are of greater importance as HML-2 LTRs are thought to function independently of TATA box and initiator elements [62] and a substantial loss in promoter activity was only seen when all GC boxes were removed from truncation constructs (Figure 5D). Our data agree with previous observations that the ubiquitous transcription factors Sp1 and Sp3, which bind to GC boxes found in promoter sequences, play a large role in regulating HML-2 promoter activity [62,63].

HML-2 LTR promoter activity is cell type-specific and depends on a number of factors including epigenetics, transcription factor binding and, possibly, proximity to other expressed genes [3,61,69]. For example, the highly expressed 22q11.21 provirus is situated very close to the expressed cellular gene PRODH (Figure 4B), which may give the LTR access to transcriptional machinery and affect its transcription, although the reverse has also been proposed [60]. Similar LTR Hs elements (1p31.1a, 11q22.1 and 12q13.2) that retain promoter motifs but are located in less actively transcribed regions do not appear to be highly expressed in Tera-1 cells based on FPKM; however, these LTRs also do not show high promoter activity in *in vitro* assay, which should have alleviated epigenetic repression of the LTR if present (Figure S5). It is possible that there are other promoter elements present on active LTRs that allow for their expression in Tera-1 cells beyond the GC and TATA boxes analyzed in this study. Along the same lines, the 22q11.21 5’ LTR Hs showed high activity in promoter assays in Tera-1 cells (Figure 5B), but very little activity in breast cancer cell lines (Figure S6). It is likely that expression results from a disease-state or tissue-specific factor acting on the LTR, especially given that the LTR Hs driven transcription of the LTR 5B provirus on 22q11.23 coincides with a known lincRNA of unknown function annotated in hg19 [59]. Its expression is highest in prostate tissue, testes and ovaries, likely reflecting the tissue-specific transcriptional regulation of the ancestral HML-2 virus.

Based on the predicted ORFs for the expressed proviruses (Figure 2B), the majority of the expressed HML-2 transcripts encode *gag* (~55%), including full-length and truncated forms, with *env* (~4%) and *pol* ORF (~5%) represented at much lower levels (Figure 2C). Based on preliminary analysis, the full-length 22q11.21 Gag has functional protease cleavage sites, whereas in the truncated 22q11.23 Gag these sites are mutated [70]. Electron microscopy of Tera-1 virions shows immature particles budding from cells [12], however the relative contributions of ineffective Gag processing, co-packaging of full-length and truncated Gag and/or lack of functional protease to this phenomenon were not determined. In terms of morphology, Tera-1 virions infrequently show Env studding [12], an observation consistent with our RNASeq data, which show only ~4% of HML-2 transcripts, originating from two expressed proviruses, to be capable of expressing Env protein. In fact, western blotting for TM shows that Env protein in Tera-1 cells is not detectable (Figure S7). The Env protein produced from the 7p22.1 tandem duplicated provirus which contributed 70% of the possible *env* transcripts, has been shown to be functional, however Env encoded by the 6q14.1 locus is not [71].

Tera-1 virions have not been shown to be infectious [14]. The primary packaged genome originating from the Type 1 provirus 22q11.21 has only an ORF for *gag* [13]. Although we did observe the packaging of other HML-2 genomes that could potentially be co-packaged and lead to recombination, the defective nature of the particle structure is likely to impede a proper infection cycle, thus preventing recombination and infectious virus production. Interestingly, the genomes that are selected for packaging all originate from LTR Hs proviruses that are human specific (Figure 2F, Figure 3B). In fact, genomes derived from these proviruses are preferentially selected for packaging over other highly expressed proviruses in Tera-1 cells (Figure 3). Potentially, only the recently integrated proviruses retain a functional packaging signal on their genomes that allows for their enrichment into Tera-1 virions. A packaging signal for HML-2 has not been reported; however, if consistent with other retroviruses, it is likely be present in the 5’ UTR region upstream of the *gag* initiation codon [72], and perhaps extending into *gag*. A result that helps elucidate necessary elements for packaging is the absence of transcripts of provirus 12q24.11 from virions, even though this recently integrated provirus is expressed in Tera-1 cells (Figure 2B,E). While 12q24.11 has *gag* leader sequence, the total provirus only retains sequence from the start of the 5’ LTR into the first ~400 nucleotides of *gag*. Potentially, sequence beyond the beginning of *gag* is necessary for the proper structure of the HML-2 packaging signal. 12q24.11 also has 5 polymorphisms in its *gag* leader in comparison to the highly packaged 22q11.21 provirus that might impair the packaging motif. The roles of these differences remain to be tested. The observation that highly expressed cellular RNAs appear to be nonspecifically packaged into HML-2 virions is consistent with other retroviruses [73].

The biological significance of HML-2 transcription in Tera-1 cells, and even more remarkably, their virion production, is not clear. Likely, HML-2 expression in these cells is purely a relic of LTR responsiveness to the transcriptional environment. Thus, the production of virions in these cells is coincidental to the proviruses with responsive LTR motifs. By analysis of HML-2 proviral transcription and selective packaging into virions, we should be able to elucidate elements of HML-2 biology that were relevant to their lifecycle as infectious retroviruses. Furthermore, in utilizing a high throughput approach independent of most PCR limitations, we can assess the full scope of HML-2 expression in the context of the cell. In the future, application of HML-2 profiling to both healthy and diseased tissues will be of great use to help elucidate the effect of HML-2 expression in the human host.

## Supplementary Files

Supplementary File 1

## References

[1] Boeke J.D., Stoye J.S., Coffin J.M., Hughes S.H., Varmus H.E. (1997). Retrotransposons, Endogenous Retroviruses, and the Evolution of Retroelements. Retroviruses.

[2] Bannert N., Kurth R. (2006). The Evolutionary Dynamics of Human Endogenous Retroviral Families. Annu. Rev. Genomics Hum. Genet..

[3] Jern P., Coffin J.M. (2008). Effects of Retroviruses on Host Genome Function. Annu. Rev. Genet..

[4] Hughes J.F., Coffin J.M. (2004). Human Endogenous Retrovirus K Solo-Ltr Formation and Insertional Polymorphisms: Implications for Human and Viral Evolution. Proc. Natl. Acad. Sci. USA.

[5] Subramanian R.P., Wildschutte J.H., Russo C., Coffin J.M. (2011). Identification, Characterization, and Comparative Genomic Distribution of the Herv-K (Hml-2) Group of Human Endogenous Retroviruses. Retrovirology.

[6] Belshaw R., Dawson A.L., Woolven-Allen J., Redding J., Burt A., Tristem M. (2005). Genomewide Screening Reveals High Levels of Insertional Polymorphism in the Human Endogenous Retrovirus Family Herv-K(Hml2): Implications for Present-Day Activity. J. Virol..

[7] Marchi E., Kanapin A., Magiorkinis G., Belshaw R. (2014). Unfixed Endogenous Retroviral Insertions in the Human Population. J. Virol..

[8] Callahan R., Chiu I.M., Wong J.F., Tronick S.R., Roe B.A., Aaronson S.A., Schlom J. (1985). A New Class of Endogenous Human Retroviral Genomes. Science.

[9] Franklin G.C., Chretien S., Hanson I.M., Rochefort H., May F.E.B., Westley B.R. (1988). Expression of Human Sequences Related to Those of Mouse Mammary Tumor Virus. J. Virol..

[10] Bittner J.J. (1936). Some Possible Effects of Nursing on the Mammary Gland Tumor Incidence in Mice. Science.

[11] Boller K., Konig H., Sauter M., Mueller-Lantzsch N., Lower R., Lower J., Kurth R. (1993). Evidence That Herv-K Is the Endogenous Retrovirus Sequence That Codes for the Human Teratocarcinoma-Derived Retrovirus Htdv. Virology.

[12] Bieda K., Hoffmann A., Boller K. (2001). Phenotypic Heterogeneity of Human Endogenous Retrovirus Particles Produced by Teratocarcinoma Cell Lines. J. Gen. Virol..

[13] Ruprecht K., Ferreira H., Flockerzi A., Wahl S., Sauter M., Mayer J., Mueller-Lantzsch N. (2008). Human Endogenous Retrovirus Family Herv-K(Hml-2) Rna Transcripts Are Selectively Packaged into Retroviral Particles Produced by the Human Germ Cell Tumor Line Tera-1 and Originate Mainly from a Provirus on Chromosome 22q11.21. J. Virol..

[14] Lower R., Lower J., Frank H., Harzmann R., Kurth R. (1984). Human Teratocarcinomas Cultured *in Vitro* Produce Unique Retrovirus-Like Viruses. J. Gen. Virol..

[15] Goedert J.J., Sauter M.E., Jacobson L.P., Vessella R.L., Hilgartner M.W., Leitman S.F., Fraser M.C., Mueller-Lantzsch N.G. (1999). High Prevalence of Antibodies against Herv-K10 in Patients with Testicular Cancer but Not with Aids. Cancer Epidemiol. Biomark. Prev..

[16] Sauter M., Schommer S., Kremmer E., Remberger K., Dölken G., Lemm I., Buck M., Best B., Neumann-Haefelin D., Mueller-Lantzsch N. (1995). Human Endogenous Retrovirus K10: Expression of Gag Protein and Detection of Antibodies in Patients with Seminomas. J. Virol..

[17] Kleiman A., Senyuta N., Tryakin A., Sauter M., Karseladze A., Tjulandin S., Gurtsevitch V., Mueller-Lantzsch N. (2004). Herv-K(Hml-2) Gag/Env Antibodies as Indicator for Therapy Effect in Patients with Germ Cell Tumors. Int. J. Cancer.

[18] Kurth R., Bannert N. (2010). Beneficial and Detrimental Effects of Human Endogenous Retroviruses. Int. J. Cancer.

[19] Boese A., Sauter M., Mueller-Lantzsch N. (2000). A Rev-Like Nes Mediates Cytoplasmic Localization of Herv-K Corf. FEBS Lett..

[20] Hohn O., Hanke K., Bannert N. (2013). Herv-K(Hml-2), the Best Preserved Family of Hervs: Endogenization, Expression, and Implications in Health and Disease. Front. Oncol..

[21] Patience C., Simpson G.R., Colletta A.A., Welch H.M., Weiss R.A., Boyd M.T. (1996). Human Endogenous Retrovirus Expression and Reverse Transcriptase Activity in the T47d Mammary Carcinoma Cell Line. J. Virol..

[22] Etkind P.R., Lumb K., Du J., Racevskis J. (1997). Type 1 Herv-K Genome Is Spliced into Subgenomic Transcripts in the Human Breast Tumor Cell Line T47d. Virology.

[23] Wang-Johanning F., Frost A.R., Johanning G.L., Khazaeli M.B., LoBuglio A.F., Shaw D.R., Strong T.V. (2001). Expression of Human Endogenous Retrovirus K Envelope Transcripts in Human Breast Cancer. Clin. Cancer Res..

[24] Wang-Johanning F., Frost A.R., Jian B., Epp L., Lu D.W., Johanning G.L. (2003). Quantitation of Herv-K Env Gene Expression and Splicing in Human Breast Cancer. Oncogene.

[25] Wang-Johanning F., Radvanyi L., Rycaj K., Plummer J.B., Yan P., Sastry K.J., Piyathilake C.J., Hunt K.K., Johanning G.L. (2008). Human Endogenous Retrovirus K Triggers an Antigen-Specific Immune Response in Breast Cancer Patients. Cancer Res..

[26] Wang-Johanning F., Rycaj K., Plummer J.B., Li M., Yin B., Frerich K., Garza J.G., Shen J., Lin K., Yan P. (2012). Immunotherapeutic Potential of Anti-Human Endogenous Retrovirus-K Envelope Protein Antibodies in Targeting Breast Tumors. J. Natl. Cancer Inst..

[27] Buscher K., Hahn S., Hofmann M., Trefzer U., Ozel M., Sterry W., Lower J., Lower R., Kurth R., Denner J. (2006). Expression of the Human Endogenous Retrovirus-K Transmembrane Envelope, Rec and Np9 Proteins in Melanomas and Melanoma Cell Lines. Melanoma Res..

[28] Muster T., Waltenberger A., Grassauer A., Hirschl S., Caucig P., Romirer I., Fodinger D., Seppele H., Schanab O., Magin-Lachmann C. (2003). An Endogenous Retrovirus Derived from Human Melanoma Cells. Cancer Res..

[29] Contreras-Galindo R., Gonzalez M., Almodovar-Camacho S., Gonzalez-Ramirez S., Lorenzo E., Yamamura Y. (2006). A New Real-Time-Rt-Pcr for Quantitation of Human Endogenous Retroviruses Type K (Herv-K) Rna Load in Plasma Samples: Increased Herv-K Rna Titers in Hiv-1 Patients with Haart Non-Suppressive Regimens. J. Virol. Methods.

[30] Contreras-Galindo R., Kaplan M.H., Markovitz D.M., Lorenzo E., Yamamura Y. (2006). Detection of Herv-K(Hml-2) Viral Rna in Plasma of Hiv Type 1-Infected Individuals. AIDS Res. Hum. Retrovir..

[31] Contreras-Galindo R., Lopez P., Velez R., Yamamura Y. (2007). Hiv-1 Infection Increases the Expression of Human Endogenous Retroviruses Type K (Herv-K) *in Vitro*. AIDS Res. Hum. Retrovir..

[32] Ormsby C.E., Sengupta D., Tandon R., Deeks S.G., Martin J.N., Jones R.B., Ostrowski M.A., Garrison K.E., Vazquez-Perez J.A., Reyes-Teran G. (2012). Human Endogenous Retrovirus Expression is Inversely Associated with Chronic Immune Activation in Hiv-1 Infection. PLoS One.

[33] Contreras-Galindo R., Kaplan M.H., Contreras-Galindo A.C., Gonzalez-Hernandez M.J., Ferlenghi I., Giusti F., Lorenzo E., Gitlin S.D., Dosik M.H., Yamamura Y. (2012). Characterization of Human Endogenous Retroviral Elements in the Blood of Hiv-1-Infected Individuals. J. Virol..

[34] Bhardwaj N., Maldarelli F., Mellors J., Coffin J.M. (2014). HIV-1 Infection Leads to Increased Transcription of Herv-K (Hml-2) Proviruses *in vivo* but Not to Increased Virion Production. J. Virol..

[35] Palmer S., Kearney M., Maldarelli F., Halvas E.K., Bixby C.J., Bazmi H., Rock D., Falloon J., Davey R.T., Dewar R.L. (2005). Multiple, Linked Human Immunodeficiency Virus Type 1 Drug Resistance Mutations in Treatment-Experienced Patients Are Missed by Standard Genotype Analysis. J. Clin. Microbiol..

[36] Santoni F.A., Guerra J., Luban J. (2012). Herv-H Rna is Abundant in Human Embryonic Stem Cells and a Precise Marker for Pluripotency. Retrovirology.

[37] Schmitt K., Richter C., Backes C., Meese E., Ruprecht K., Mayer J. (2013). Comprehensive Analysis of Human Endogenous Retrovirus Group Herv-W Locus Transcription in Multiple Sclerosis Brain Lesions by High-Throughput Amplicon Sequencing. J. Virol..

[38] Brinzevich D., Young G.R., Sebra R., Ayllon J., Maio S.M., Deikus G., Chen B.K., Fernandez-Sesma A., Simon V., Mulder L.C. (2014). Hiv-1 Interacts with Human Endogenous Retrovirus K (Hml-2) Envelopes Derived from Human Primary Lymphocytes. J. Virol..

[39] Gonzalez-Hernandez M.J., Cavalcoli J.D., Sartor M.A., Contreras-Galindo R., Meng F., Dai M., Dube D., Saha A.K., Gitlin S.D., Omenn G.S. (2014). Regulation of the Herv-K (Hml-2) Transcriptome by the HIV-1 Tat Protein. J. Virol..

[40] Simpson G.R., Patience C., Löwer R., Tönjes R.R., Moore H.D.M., Weiss R.A., Boyd M.T. (1996). Endogenous D-Type (Herv-K) Related Sequences Are Packaged into Retroviral Particles in the Placenta and Possess Open Reading Frames for Reverse Transcriptase. Virology.

[41] Li M.D., Bronson D.L., Lemke T.D., Faras A.J. (1995). Restricted Expression of New Herv-K Members in Human Teratocarcinoma Cells. Virology.

[42] Bolger A.M., Lohse M., Usadel B. (2014). Trimmomatic: A Flexible Trimmer for Illumina Sequence Data. Bioinformatics.

[43] Langmead B., Salzberg S.L. (2012). Fast Gapped-Read Alignment with Bowtie 2. Nat. Methods.

[44] Kim D., Pertea G., Trapnell C., Pimentel H., Kelley R., Salzberg S.L. (2013). Tophat2: Accurate Alignment of Transcriptomes in the Presence of Insertions, Deletions and Gene Fusions. Genome Biol..

[45] Li H., Handsaker B., Wysoker A., Fennell T., Ruan J., Homer N., Marth G., Abecasis G., Durbin R., Genome Project Data Processing S. (2009). The Sequence Alignment/Map Format and Samtools. Bioinformatics.

[46] Roberts A., Trapnell C., Donaghey J., Rinn J.L., Pachter L. (2011). Improving Rna-Seq Expression Estimates by Correcting for Fragment Bias. Genome Biol..

[47] Mortazavi A., Williams B.A., McCue K., Schaeffer L., Wold B. (2008). Mapping and Quantifying Mammalian Transcriptomes by Rna-Seq. Nat. Methods.

[48] Sayers E.W., Barrett T., Benson D.A., Bolton E., Bryant S.H., Canese K., Chetvernin V., Church D.M., DiCuccio M., Federhen S. (2011). Database Resources of the National Center for Biotechnology Information. Nucleic Acids Res..

[49] Thorvaldsdottir H., Robinson J.T., Mesirov J.P. (2013). Integrative Genomics Viewer (Igv): High-Performance Genomics Data Visualization and Exploration. Brief. Bioinform..

[50] Kent W.J., Sugnet C.W., Furey T.S., Roskin K.M., Pringle T.H., Zahler A.M., Haussler D. (2002). The Human Genome Browser at Ucsc. Genome Res..

[51] Huang W., Li L., Myers J.R., Marth G.T. (2012). Art: A Next-Generation Sequencing Read Simulator. Bioinformatics.

[52] Tamura K., Stecher G., Peterson D., Filipski A., Kumar S. (2013). Mega6: Molecular Evolutionary Genetics Analysis Version 6.0. Mol. Biol. Evol..

[53] Karolchik D., Hinrichs A.S., Furey T.S., Roskin K.M., Sugnet C.W., Haussler D., Kent W.J. (2004). The Ucsc Table Browser Data Retrieval Tool. Nucleic Acids Res..

[54] Untergasser A., Cutcutache I., Koressaar T., Ye J., Faircloth B.C., Remm M., Rozen S.G. (2012). Primer3—New Capabilities and Interfaces. Nucleic Acids Res..

[55] Ono M., Kawakami M., Takezawa T. (1987). A Novel Human Nonviral Retroposon Derived from an Endogenous Retrovirus. Nucleic Acids Res..

[56] Lee Y.N., Malim M.H., Bieniasz P.D. (2008). Hypermutation of an Ancient Human Retrovirus by Apobec3g. J. Virol..

[57] Armbruester V., Sauter M., Krautkraemer E., Meese E., Kleiman A., Best B., Roemer K., Mueller-Lantzsch N. (2002). A Novel Gene from the Human Endogenous Retrovirus K Expressed in Transformed Cells. Clin. Cancer Res..

[58] Löwer R., Tönjes R.R., Korbmacher C., Kurth R., Löwer J. (1995). Identification of a Rev-Related Protein by Analysis of Spliced Transcripts of the Human Endogenous Retroviruses Htdv/Herv-K. J. Virol..

[59] Cabili M.N., Trapnell C., Goff L., Koziol M., Tazon-Vega B., Regev A., Rinn J.L. (2011). Integrative Annotation of Human Large Intergenic Noncoding Rnas Reveals Global Properties and Specific Subclasses. Genes Dev..

[60] Suntsova M., Gogvadze E.V., Salozhin S., Gaifullin N., Eroshkin F., Dmitriev S.E., Martynova N., Kulikov K., Malakhova G., Tukhbatova G. (2013). Human-Specific Endogenous Retroviral Insert Serves as an Enhancer for the Schizophrenia-Linked Gene Prodh. Proc. Natl. Acad. Sci. USA.

[61] Ruda V.M., Akopov S.B., Trubetskoy D.O., Manuylov N.L., Vetchinova A.S., Zavalova L.L., Nikolaev L.G., Sverdlov E.D. (2004). Tissue Specificity of Enhancer and Promoter Activities of a Herv-K(Hml-2) Ltr. Virus Res..

[62] Manghera M., Douville R.N. (2013). Endogenous Retrovirus-K Promoter: A Landing Strip for Inflammatory Transcription Factors?. Retrovirology.

[63] Fuchs N.V., Kraft M., Tondera C., Hanschmann K.M., Lower J., Lower R. (2011). Expression of the Human Endogenous Retrovirus (Herv) Group Hml-2/Herv-K Does Not Depend on Canonical Promoter Elements but Is Regulated by Transcription Factors Sp1 and Sp3. J. Virol..

[64] Kovalskaya E., Buzdin A., Gogvadze E., Vinogradova T., Sverdlov E. (2006). Functional Human Endogenous Retroviral Ltr Transcription Start Sites Are Located between the R and U5 Regions. Virology.

[65] Ruprecht K., Mayer J., Sauter M., Roemer K., Mueller-Lantzsch N. (2008). Endogenous Retroviruses and Cancer. Cell. Mol. Life Sci..

[66] Van der Kuyl A.C. (2012). Hiv Infection and Herv Expression: A Review. Retrovirology.

[67] Seifarth W., Frank O., Zeilfelder U., Spiess B., Greenwood A.D., Hehlmann R., Leib-Mosch C. (2005). Comprehensive Analysis of Human Endogenous Retrovirus Transcriptional Activity in Human Tissues with a Retrovirus-Specific Microarray. J. Virol..

[68] Flockerzi A., Ruggieri A., Frank O., Sauter M., Maldener E., Kopper B., Wullich B., Seifarth W., Muller-Lantzsch N., Leib-Mosch C. (2008). Expression Patterns of Transcribed Human Endogenous Retrovirus Herv-K(Hml-2) Loci in Human Tissues and the Need for a Herv Transcriptome Project. BMC Genomics.

[69] Lavie L., Kitova M., Maldener E., Meese E., Mayer J. (2005). Cpg Methylation Directly Regulates Transcriptional Activity of the Human Endogenous Retrovirus Family Herv-K(Hml-2). J. Virol..

[70] George M., Schwecke T., Beimforde N., Hohn O., Chudak C., Zimmermann A., Kurth R., Naumann D., Bannert N. (2011). Identification of the Protease Cleavage Sites in a Reconstituted Gag Polyprotein of an Herv-K(Hml-2) Element. Retrovirology.

[71] Dewannieux M., Blaise S., Heidmann T. (2005). Identification of a Functional Envelope Protein from the Herv-K Family of Human Endogenous Retroviruses. J. Virol..

[72] D’Souza V., Summers M.F. (2005). How Retroviruses Select Their Genomes. Nat. Rev. Microbiol..

[73] Rulli S.J., Hibbert C.S., Mirro J., Pederson T., Biswal S., Rein A. (2007). Selective and Nonselective Packaging of Cellular Rnas in Retrovirus Particles. J. Virol..

