# Supplementary material for: Differential Expression of HERV-K (HML-2) Proviruses in Cells and Virions of the Teratocarcinoma Cell Line Tera-1
Source: Viruses. 2015 Mar 4;7(3):939–68. doi: 10.3390/v7030939 (PMC4379556; doi:10.3390/v7030939)
Supplement: Supplementary File 1 [file viruses-07-00939-s001.zip › v7030939-supplementary-1.pdf]

Supplementary Material

1. Figure Legends

Figure S1. RNASeq analysis of HML-2 expression in Tera-1 cells and virions as determined by alignment to an HML-2 reference genome

RNASeq reads originating from Tera-1 cells and virions were aligned to the HML-2 reference genome and analyzed according to the Unstranded, Unique Only analysis (A-B). For the duplicated proviruses at 7p22.1a/b (tandem) and 8p23.1b/c/d (non-K113), the FPKM values for individual duplicated proviruses were added together to represent the duplications as one locus since reads aligning to a duplicated provirus may have originated from any of the proviral duplications. For C-D, a modified HML-2 reference genome was created that represented duplicated proviruses with only one locus for alignment. This was performed for the potentially underrepresented loci at 7p22.1**a**/b (LTR Hs), 1p36.21a/**b**/c (LTR 5B), Xq28a/**b** (LTR 5B), Yq11.23**a**/b (LTR 5B) and 8p23.1b/**c**/d (LTR 5A) identified in the in-silico simulation (Fig 1B). The locus included for alignment is shown in bold. RNASeq reads originating from Tera-1 cells and virions were then aligned to this modified HML-2 reference genome without duplications and analyzed according to the Unstranded, Unique Only analysis (C-D). Based off of the stranded alignment to hg19 (Fig 1A), the expression values for 7q34, 11q12.3, 11q23.3 and the 8p23.1 duplicated locus seen in A and C can be attributed to minus sense transcription. The (*) symbols refer to proviruses predicted to be underrepresented by 15% or more based on an in silico simulation (as in Fig 1).

Figure S2. Full provirus neighbor-joining tree of HML-2 loci expressed in Tera-1 cells

A neighbor-joining tree of the HML-2 proviruses expressed in Tera-1 cells was generated using the full sequence of each provirus. The p-distance method was used to calculate distance and bootstrap values are indicated (1000 replicates). Proviruses with (*) were predicted to be underrepresented in the in-silico analysis (Fig 1B). Solid squares indicate those proviruses (11q23.3 and 11q12.3) whose transcripts are minus sense and initiate from a neighboring transcription unit. Solid diamonds indicate those proviruses (4p16.3a and 22q11.23) whose transcription is plus sense, but originate from a neighboring transcription unit and not the corresponding 5' LTR. Proviruses 19p12d and 1p31.1a are boxed to indicate that they were only detected as expressed highly in the HML-2 reference genome alignment.

Figure S3. UCSC genome browser view of RNASeq reads aligned to provirus 22q11.23

RNASeq reads from Tera-1 cells were aligned to the hg19 build of the human genome, filtered for Unique Only reads and a BEDfile of the alignment was uploaded to the UCSC genome browser as a custom track. A screenshot of the UCSC genome browser around LTR 5B provirus 22q11.23 (chr22: 23879930-23890615) is shown. A cartoon is depicted above the UCSC Genome browser view to clearly indicate the LTR Hs element driving transcription, the 22q11.23 provirus, the splice site observed and the lincRNA annotated in the genome that overlaps this locus. The black arrow indicates the start site and direction of transcription observed in Tera-1 cells and the red arrow points to a region of the provirus that was not covered after the Unique Only filtering. The UCSC Genome browser view

shows the Repeat Masker annotations and also the lincRNA highly expressed in the testes (lincRNA accession number: TCONS_l2_00017644). Of note, an AluY element is inserted at the end of *env* in the 22q11.23 provirus and a MER11A element inserted into its 3' LTR appears to be the site of transcriptional termination. A key depicting the symbols used for reads crossing splice junctions and reads that do not is shown below the screenshot.

Figure S4. UCSC genome browser view of RNASeq reads aligned to provirus 22q11.21 before and after Unique Only filtering

RNASeq reads from Tera-1 cells were aligned to the hg19 build of the human genome, and were left Unfiltered (A) or filtered for Unique Only reads (B). BEDfiles of the alignments were uploaded to the UCSC genome browser as custom tracks. Screenshots of the UCSC genome browser around LTR Hs provirus 22q11.21 (chr22: 18926187-18935307) are shown. A cartoon is depicted above the UCSC Genome browser view to indicate the distinct expression of the 22q11.21 provirus from the nearby PRODH gene. The black arrow indicates the start sites and direction of transcription for 22q11.21 and PRODH observed in Tera-1 cells. The red arrow points to a region of the provirus that exhibited poor read coverage in the Unfiltered and Unique Only alignments. The UCSC Genome browser view shows the Repeat Masker annotations and the transcript annotation for the gene PRODH. A key depicting the symbols used for reads crossing splice junctions and reads that do not is shown below the screenshots.

Figure S5. 5' LTR Promoter activity from closely related HML-2 loci not highly expressed in Tera-1 cells

Proviruses 11q22.1, 12q13.2 and 1p31.1a are closely related to highly expressed LTR Hs proviruses (Fig 1B) and retain promoter elements on their 5' LTRs. Transcripts from provirus 1p31.1a were detected in the HML-2 reference genome alignment (Fig S1A, C) but were not as abundant in the hg19 alignment (Fig 2B). Transcripts from proviruses 11q22.1 and 12q13.2 were not detected as being highly expressed in either alignment. Comparison of their transcript expression level (FPKM) from Unfiltered, Plus stranded and Unique Only, Plus stranded alignments are shown in comparison to the relative promoter expression level (RLU, normalized to SV40 promoter activity) of the proviral 5' LTRs in Tera-1 cells. All luciferase experiments were conducted in triplicates and data displays mean ± standard deviation.

Figure S6. 5' LTR Promoter activity from 22q11.21 provirus in breast cancer cell lines

The 22q11.21 provirus 5' LTR is highly active in Tera-1 cells (Fig 5B). The relative promoter expression level (RLU, normalized to SV40 promoter activity) of this 5' LTR in breast cancer cell lines is shown. All luciferase experiments were conducted in triplicates and data displays mean ± standard deviation.

Figure S7. HML-2 Env expression in Tera-1 cells

HML-2 Env expression in Tera-1 cells was analyzed by western blotting using a monoclonal antibody specific for Env TM. Sample loading was assayed via α-tubulin detection. Disparities in sample loading were quantified using ImageJ (v1.48), where the α-tubulin quantity in 293T + K-Con Env = 1 and other samples are shown as relative amounts. G355.5 (a feline astrocyte cell line), 293T and 293T

transfected with K-Con Gag-Pol serve as negative controls for HML-2 TM expression. 293T transfected with K-Con Env serves as a positive control for HML-2 TM expression.

2. Supplemental Methods

Western blotting

K-Con Gag-Pol (a consensus HML-2 Gag-Pol in pCRVI) and K-Con Env (a consensus HML-2 Env in pCRVI) plasmids were generously provided by Paul Bieniasz [55] and the SIVΔenv-GFP plasmid was generously given by David Evans [56]. K-Con Env Δ659-699 and empty vector pCRVI were generated by inverted PCR mutagenesis with Phusion polymerase (NEB, Cat# M0530S) to remove the 40 C-terminal amino acids in TM or to remove whole Env sequence from the plasmid backbone, respectively. 293T cells were transfected with 16μg of empty vector (pCRVI), K-Con Gag-Pol or K-Con Env Δ659-699 using Lipofectamine 2000 in a 1:2 ratio of DNA:reagent. 48 hours post-transfection, whole cell lysate was collected. G355.5 and Tera-1 cells were grown for 48-72 hours before harvesting whole cell lysate.

293T, G355.5 and Tera-1 cells were washed with PBS prior to the addition of chilled lysis buffer (2mM EDTA, 150 mM NaCl, 50 mM Tris-HCl pH 7.5, 1% Ipegal). Cells were scraped from cell culture plates and left on ice for 30 minutes before pelleting cellular debris to clarify the lysate. 30ug of whole cell lysate were denatured at 100°C for 10 min in Laemmli buffer with 2.5% β-mercaptoethanol (Boston BioProducts, Cat# BP-110R) and run on a 4-15% Tris-Glycine Mini-PROTEAN gel (Biorad, Cat# 456-1084) with a PrecisionPlus Protein Standard ladder (BioRad Cat# 161-0374). Samples were transferred onto PVDF membranes (Millipore, Cat# IPFL00010) and probed with anti-HML-2 TM (Austral, HERM-1811-5) in 5% non-fat dry milk (NFDM) in Tris-buffered saline (TBS; Gibco, Cat# 15567-027) with 0.025% Tween (Sigma, Cat# P1379-500). Whole cell lysates were additionally probed with anti-alpha tubulin (Sigma, Cat# T9026) in 5% NFDM in TBS-T. Membranes were stripped with Restore Plus stripping buffer (Thermo, Cat# 46430) prior to probing with other primary antibodies. Primary antibodies were detected using a goat anti-mouse-HRP antibody and membranes were visualized using Novex ECL (Invitrogen, Cat# WP20005) in a Syngene G-Box with GeneSys software (v1.2.5.0).
