# Supplementary material for: Differential Expression of HERV-K (HML-2) Proviruses in Cells and Virions of the Teratocarcinoma Cell Line Tera-1
Source: Viruses. 2015 Mar 4;7(3):939–68. doi: 10.3390/v7030939 (PMC4379556; doi:10.3390/v7030939)
Supplement: Supplementary File 1 [file viruses-07-00939-s001.zip › v7030939-supplementary-2.pdf]

Supplementary Figure 1

A

Abundance of HML-2 proviral transcripts in Tera-1 cells
(HML-2 reference alignment)

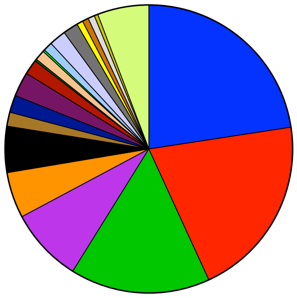

B

Abundance of HML-2 proviral transcripts in Tera-1 virions
(HML-2 reference alignment)

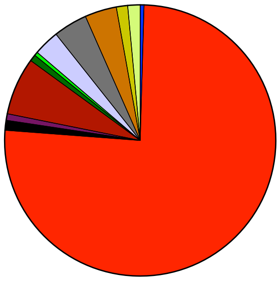

C

Abundance of HML-2 proviral transcripts in Tera-1 virions
(HML-2 reference alignment, no duplications)

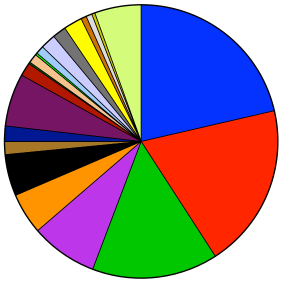

D

Abundance of HML-2 proviral transcripts in Tera-1 virions
(HML-2 reference alignment, no duplications)

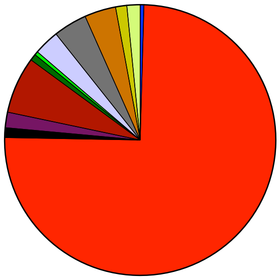

Supplementary Figure 2

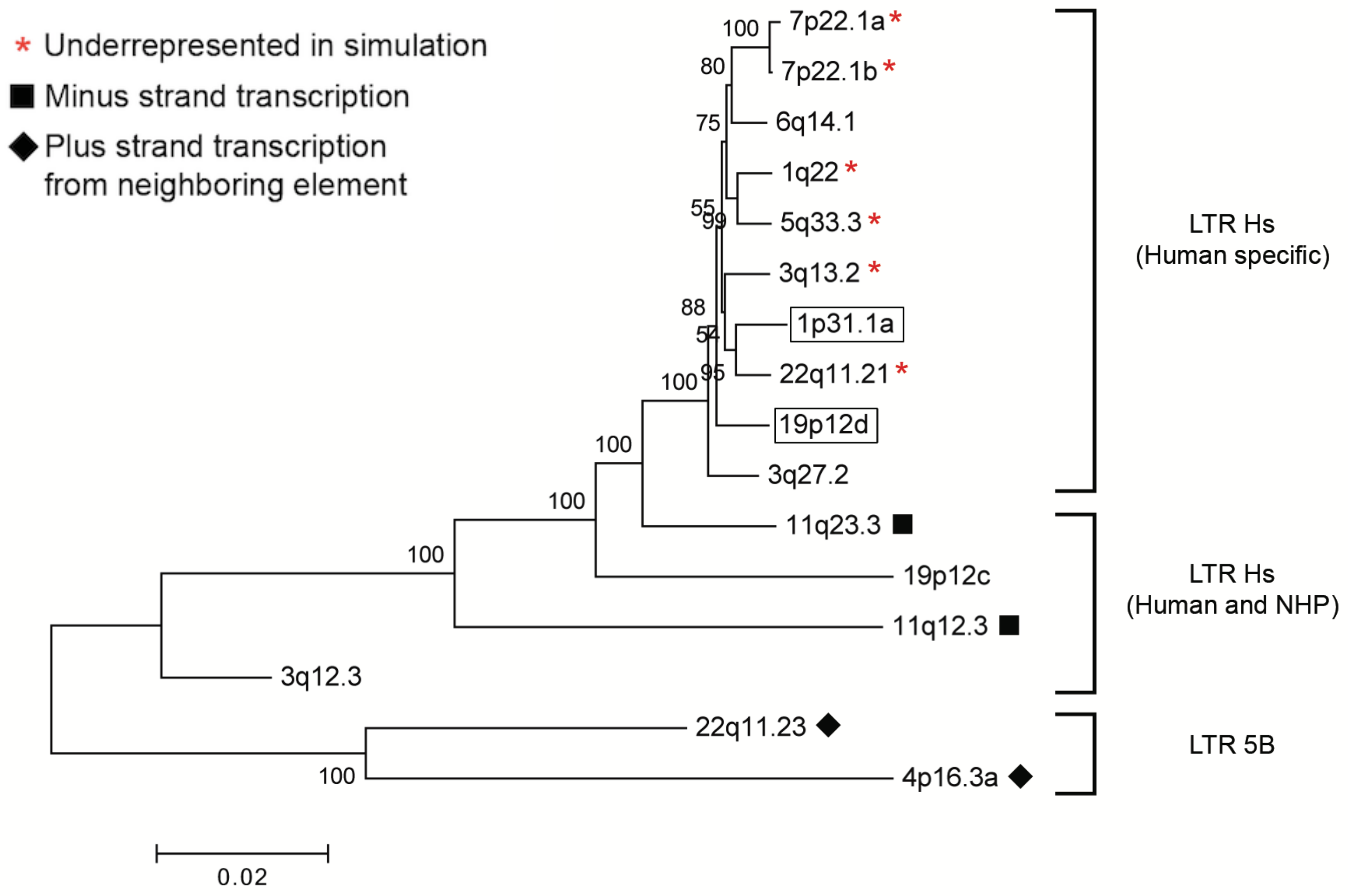

Supplementary Figure 3

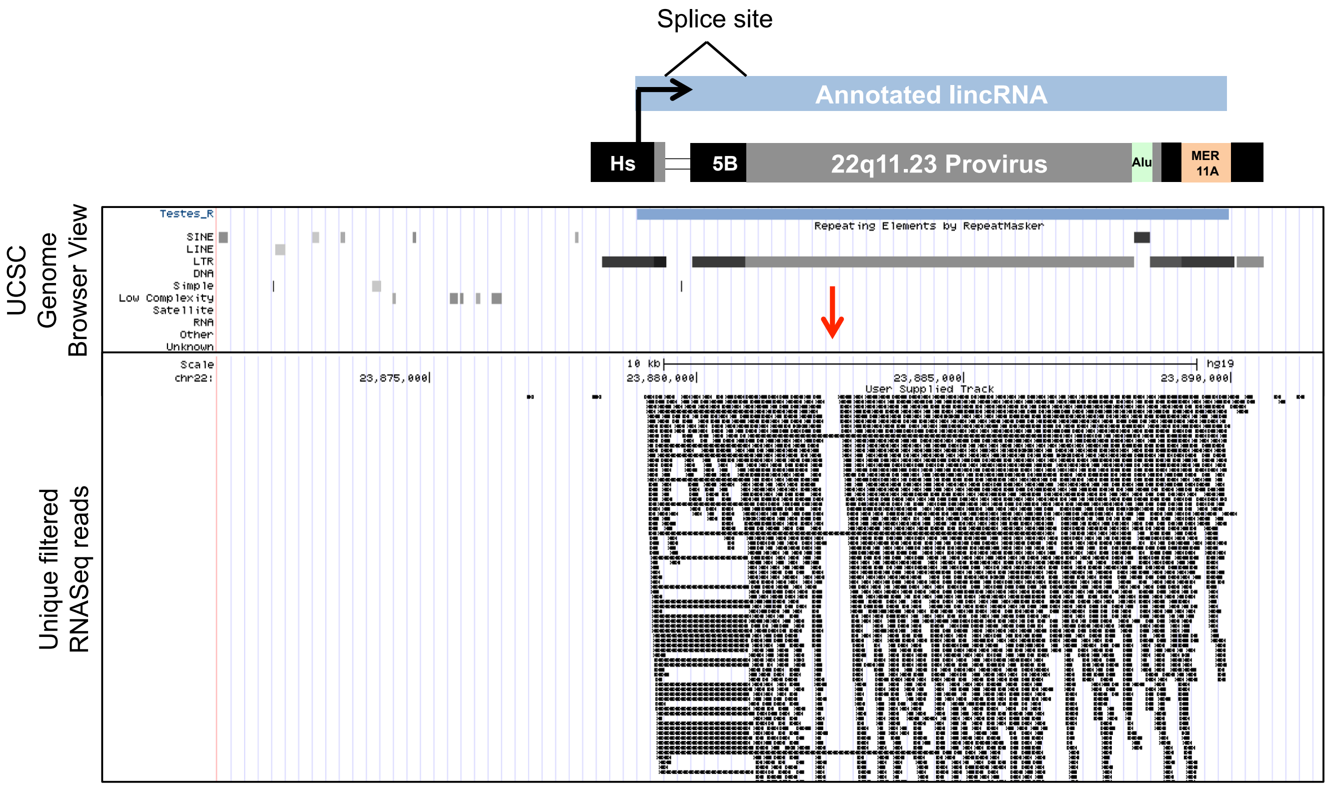

KEY:

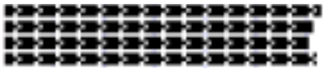

Reads crossing splice junctions
(Note: appear as one line of uniform boxes spanning 100s-1000s nt)

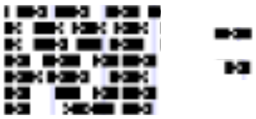

Reads that do not cross splice junctions
(Note: appear as a grouping of 1-3 boxes and span 100-301 nt)

Supplementary Figure 4

A

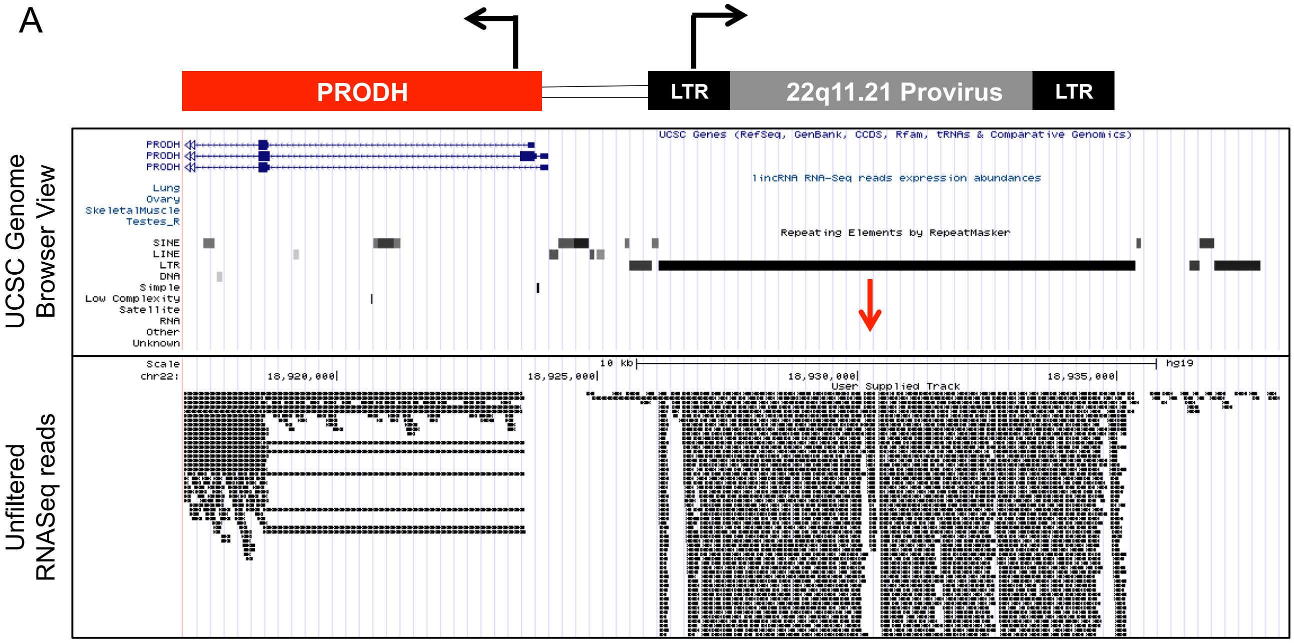

B

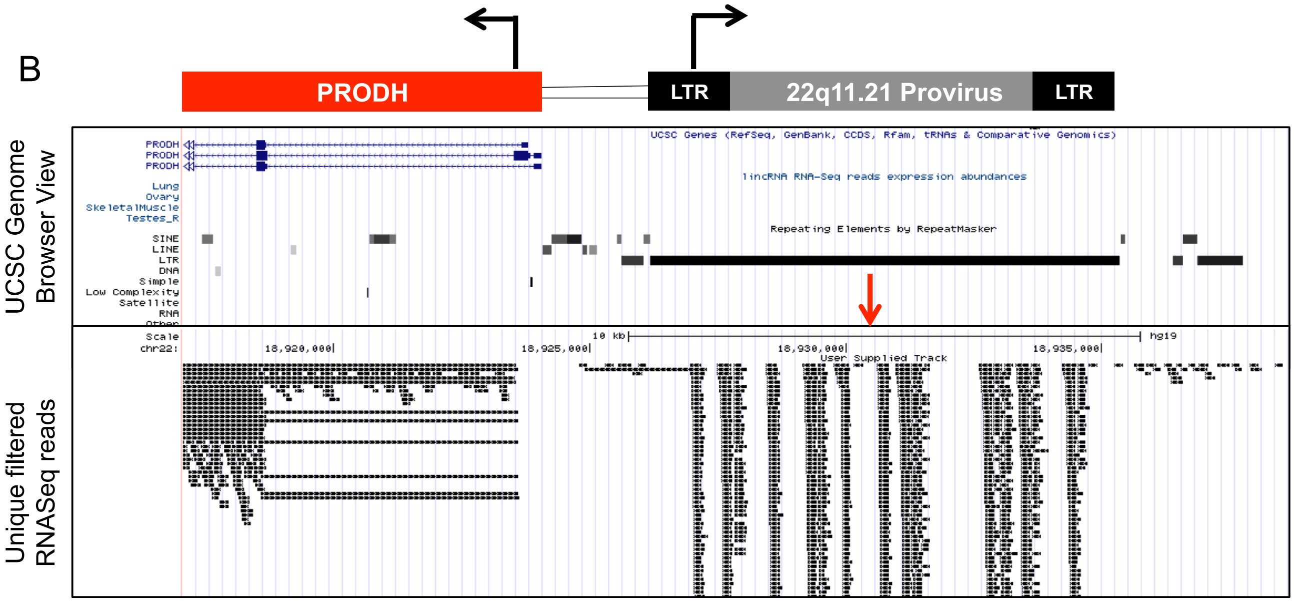

KEY:

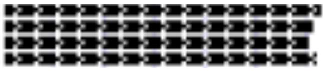

Reads crossing splice junctions
(Note: appear as one line of uniform boxes spanning 100s-1000s nt)

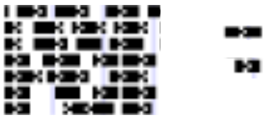

Reads that do not cross splice junctions
(Note: appear as a grouping of 1-3 boxes and span 100-301 nt)

Supplementary Figure 5

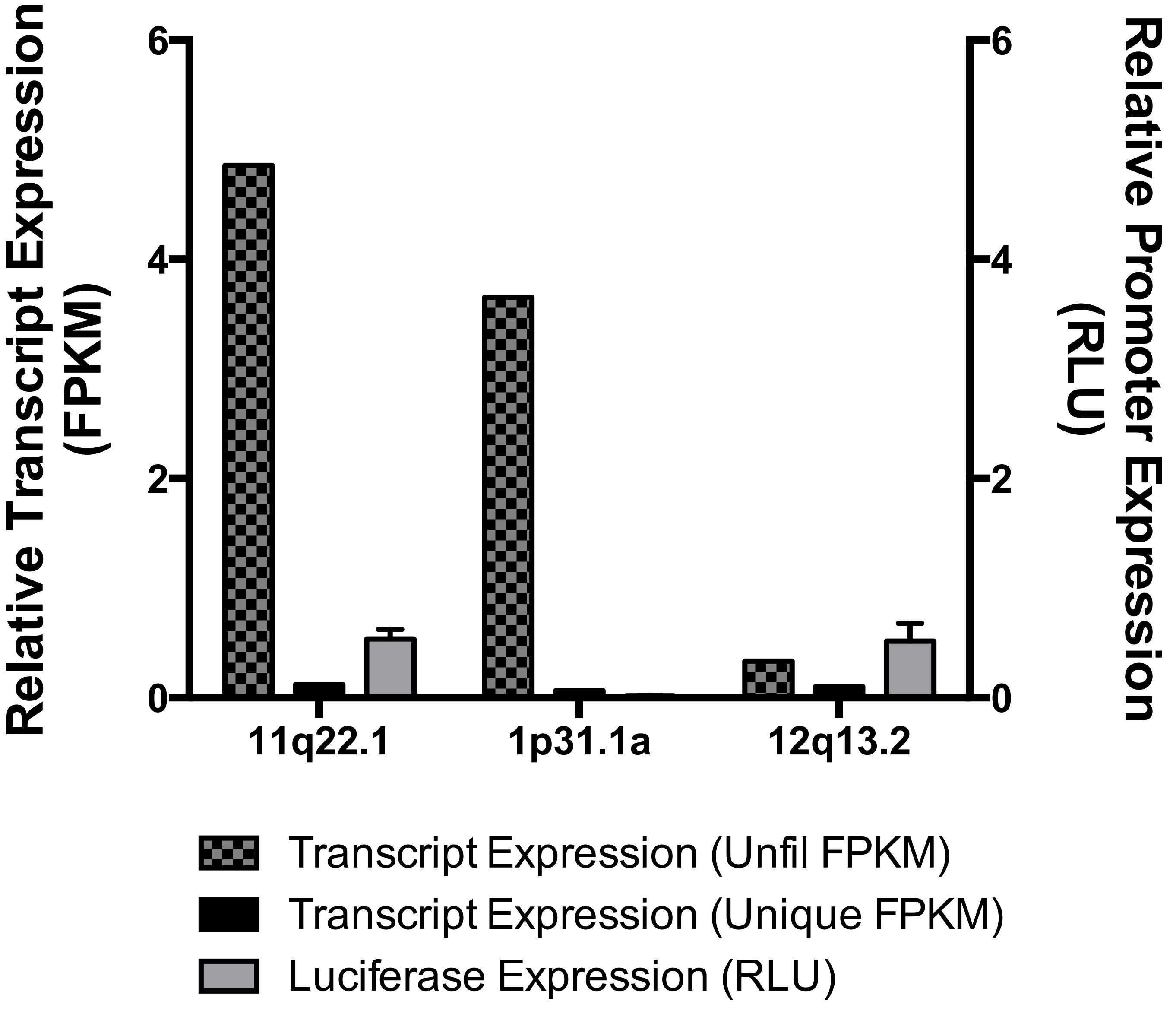

Supplementary Figure 6

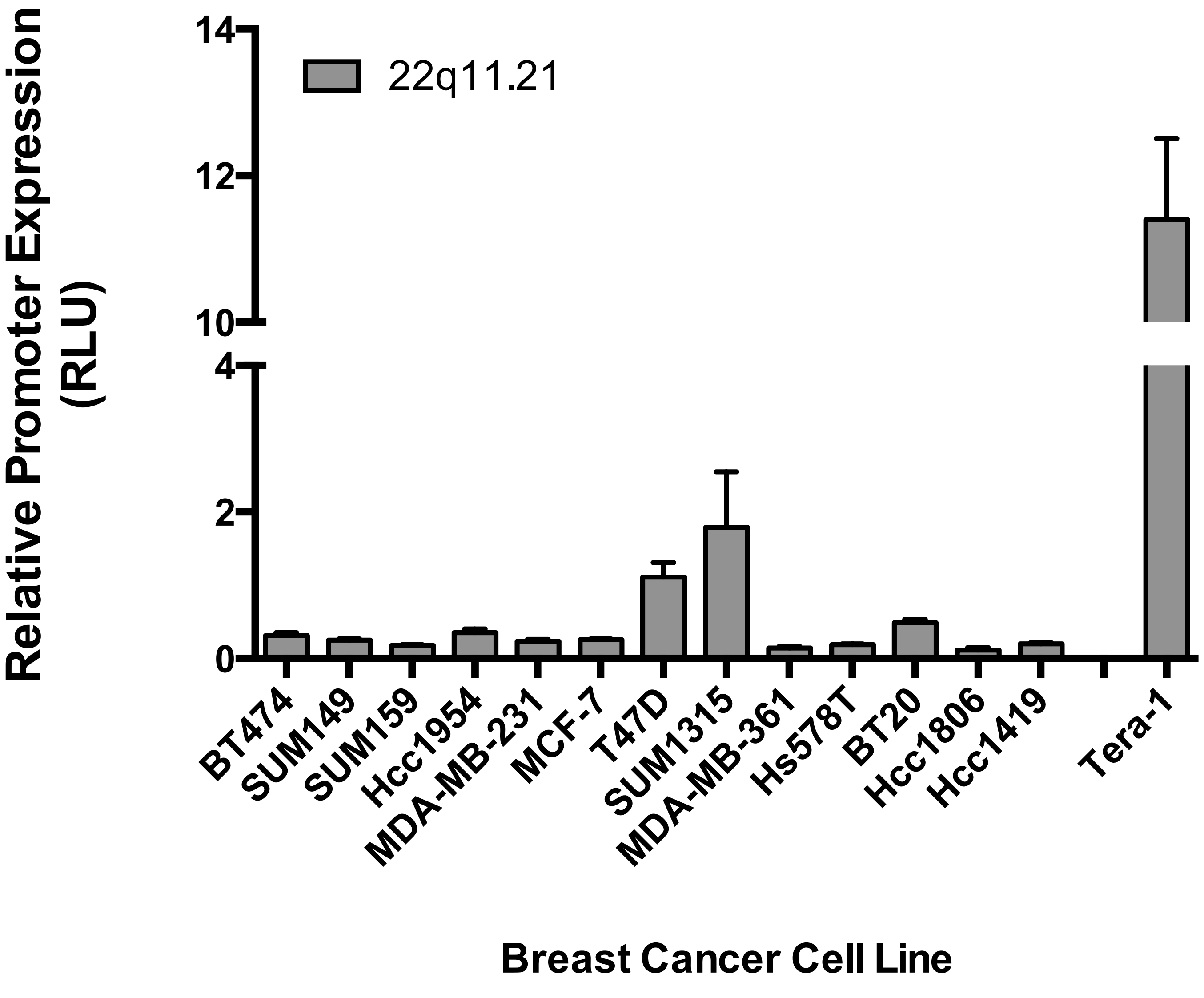

Supplementary Figure 7

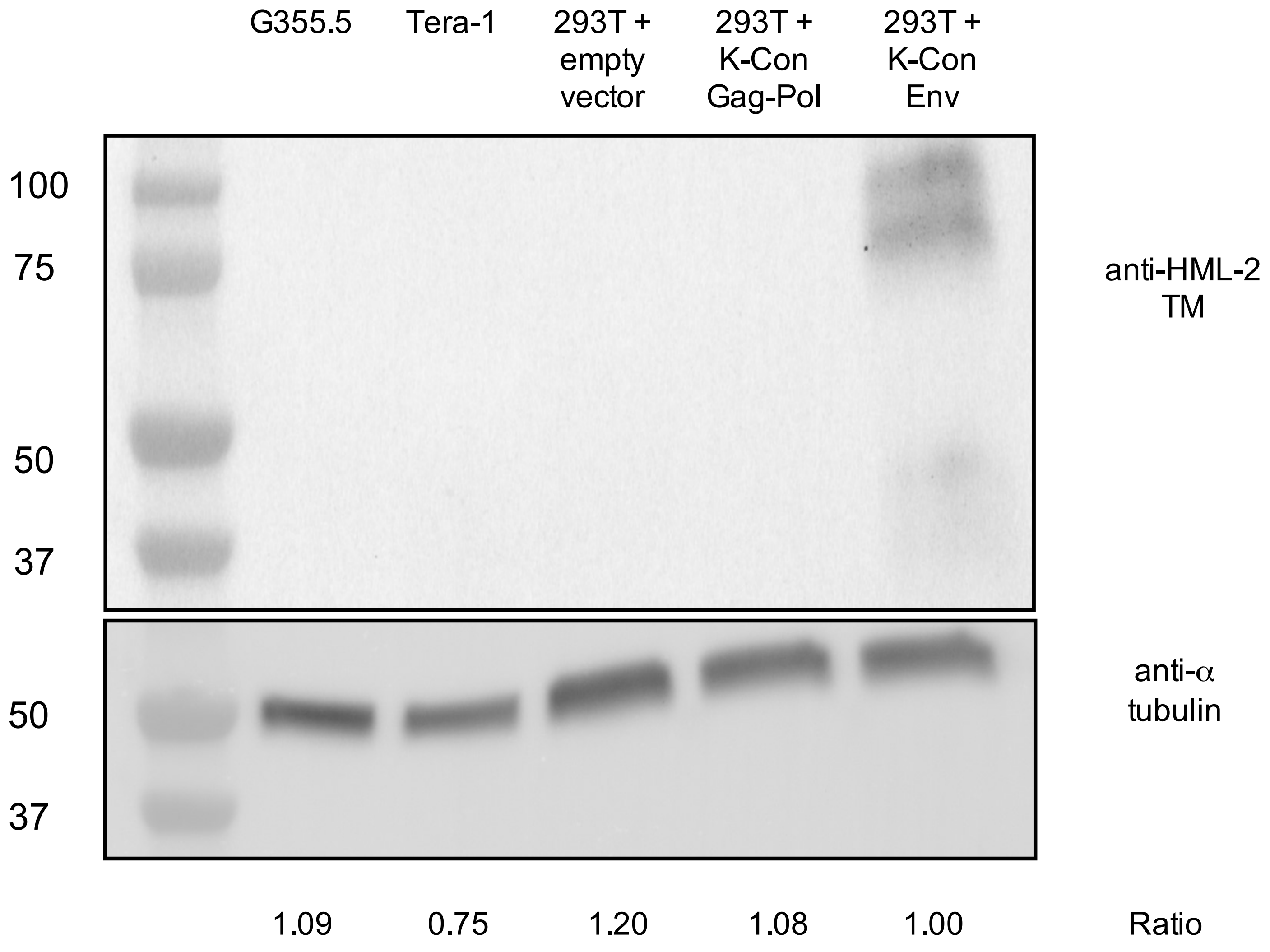

Table S1. Primers used to amplify 5' LTRs of HML-2 proviruses.

Provirus	Chromosomal Location (hg19)	Primer Sequences
22q11.21	chr22:18,926,187-18,927,154	F: 5'-ATTATAGGTACCTGCCTCAACCTCCCAAGTAG-3'<br>R: 5'-ATTATAGAGCTCCGGCTCTGCTACATATTCGC-3'
3q12.3	chr3:101,410,737-101,411,705	F: 5'-ATTATAGGTACCAAGGAGGCTGAGCAGATGAG-3'<br>R: 5'-ATTATTAAGCTTTTCCAGGGGCATCAGAAACT-3'
1q22	chr1:155,604,669-155,605,636	F: 5'-ATTATAGAGCTCCGTTGACTGAGCCATTACCG-3'<br>R: 5'-ATTATAGGTACCTAAAATCCAGCAGCCCAGGA-3'
7p22.1a	chr7:4,630,561-4,631,528	F: 5'-ATTATAGAGCTCCGTTGACTGAGCCATTACCG-3'<br>R: 5'-ATTATAGGTACCAGTCTGAGCATCACTGGGAC-3'
3q13.2	chr3:112,751,323-112,752,282	F: 5'-ATTATAGAGCTCGGGATCTCTCGTCGACTTGT-3'<br>R: 5'-ATTATAGGTACCTCCTGCCTGAGAGTTTCCAG-3'
7p22.1b	chr7:4,639,064-4,640,031	F: 5'-ATTATAGAGCTCCGTTGACTGAGCCATTACCG-3'<br>R: 5'-ATTATAGGTACCAATACCCACAGCACCCAAGA-3'
1p31.1a	chr1:75,842,771-75,843,738	F: 5'-ATTATAGGTACCGCCTCAGGAGTCGATGCTAT-3'<br>R: 5'-ATTATAGAGCTCTTCCAGGGGCATCAGAAACT-3'
22q11.23	chr22:23,879,927-23,880,916	F: 5'-ATTATAGGTACCGGAGAGAGCAGGGGTTTTCT-3'<br>R: 5'-ATTATAGAGCTCATGGCCCCAATGATTCTGGA-3'
22q11.23<br>LTR Hs	chr22:23,878,249-23,879,213	F: 5'-ATTATAGGTACCTGTTGTAAGGGGAGCTGGAG-3'<br>R: 5'-ATTATAGAGCTCTCGAGAGTCCCTTCACCCTA-3'

Table S2. Primers used to create and amplify truncated versions of 22q11.23 LTR Hs.

LTR Truncation Sites	Chromosomal Location (hg19)	Primer Sequences
1→435	chr22:23,878,249-23,878,683	F: 5’-ATTATAGGTACCGCCACTGCCATCTACTAGGA-3’<br>R: 5’-ATTATAGAGCTCTCAGCACAGACCCTTTACGG-3’
1→522	chr22:23,878,249-23,878,770	F: 5’-ATTATAGGTACCGCCACTGCCATCTACTAGGA-3’<br>R: 5’-ATTATAGAGCTCCATTCCATTGCCCAGGGATG-3’
1→740	chr22:23,878,249-23,878,988	F: 5’-ATTATAGGTACCGCCACTGCCATCTACTAGGA-3’<br>R: 5’-ATTATAGAGCTCGTAATAGTGGGGAGAGGGCC-3’
1→805	chr22:23,878,249-23,879,053	F: 5’-ATTATAGGTACCTGGGATGAACTAGAGGACGC-3’<br>R: 5’-ATTATAGAGCTCTCCCTCAGTATTTATTGATC-3’
1→815	chr22:23,878,249-23,879,063	F: 5’-ATTATAGGTACCAGTGCACAGTTCAAAACCCC-3’<br>R: 5’-ATTATAGAGCTCGTCTCTGAGTTCCCTCAGTA-3’
1→826	chr22:23,878,249-23,879,074	F: 5’-ATTATAGGTACCGGATGAACTAGAGGACGCCC-3’<br>R: 5’-ATTATAGAGCTCGCGCCGCACCGGTCTCTGAG-3’
1→847	chr22:23,878,249-23,879,095	F: 5’-ATTATAGGTACCTGGGATGAACTAGAGGACGC-3’<br>R: 5’-ATTATAGAGCTCCGCTCAGCATATGGAGGACC-3’
Full LTR (1→965)	chr22:23,878,249-23,879,213	F: 5’-ATTATAGGTACCTGTTGTAAGGGGAGCTGGAG-3’<br>R: 5’-ATTATAGAGCTCTCGAGAGTCCCTTCACCCTA-3’

Table S3. HML-2 proviruses not present in the hg19 build of the human genome.

Provirus	Chromosomal Location (hg19)	Solo LTR or Empty Site (hg19)
K105	chrUn_gl000219: 175210-176178	Solo LTR
1p31.1b	chr1: 73594980-73595948	Solo LTR
10p12.1	chr10: 27182399-27183380	Solo LTR
12q13.2	chr12: 55727215-55728183	Solo LTR
19p12b	chr19:21841536-21841542	Empty site
19p12d	chr19:22414379-22414382	Empty site

Table S4. Presence of spliced accessory transcripts in HML-2 provirus alignments

Provirus	HML-2 Type	Transcripts present in Unfiltered alignment	Transcripts present in Unique Only alignment
**22q11.23**	2	rec	rec
**22q11.21**	1	np9, hel	np9, hel
**19p12c**	1	hel	hel
**3q12.3**	1	np9, hel	np9, hel
**1q22**	1	np9, hel	none
**7p22.1**	2	rec	rec
**6q14.1**	1	rec, hel	rec
**5q33.3**	1	np9, hel	hel

The alignment of Tera-1 cell reads to the HML-2 genome was visualized using IGV. All proviruses noted in Figure 2B were examined for the presence of spliced reads. Those proviruses with spliced reads correlating to known accessory transcripts are listed in Table S4. Note that some transcripts are not present in both Unfiltered and Unique Only alignments.
